# Factors associated with accessing aged care services in Australia after approval for services: Findings from the historical cohort of the Registry of Senior Australians

**DOI:** 10.1111/ajag.12760

**Published:** 2020-01-23

**Authors:** Maria C. Inacio, Azmeraw T. Amare, Craig Whitehead, Sarah C. E. Bray, Megan Corlis, Renuka Visvanathan, Steve Wesselingh

**Affiliations:** ^1^ Healthy Ageing Research Consortium Registry of Senior Australians (ROSA) South Australian Health and Medical Research Institute (SAHMRI) Adelaide SA Australia; ^2^ Division of Health Sciences University of South Australia Adelaide SA Australia; ^3^ Adelaide Medical School University of Adelaide Adelaide SA Australia; ^4^ Department of Rehabilitation, Aged and Extended Care School of Health Sciences Flinders University Adelaide SA Australia; ^5^ Helping Hand Aged Care Adelaide SA Australia; ^6^ National Health and Medical Research Council Centre of Research Excellence in Frailty and Healthy Ageing University of Adelaide Adelaide SA Australia; ^7^ Adelaide Geriatrics Training and Research with Aged Care Centre School of Medicine University of Adelaide Adelaide SA Australia; ^8^ Aged and Extended Care Services The Queen Elizabeth Hospital Central Adelaide Local Health Network Adelaide SA Australia; ^9^ South Australian Health and Medical Research Institute (SAHMRI) Adelaide South Australia Australia

**Keywords:** aged care, healthy ageing, home care, registry, residential aged care, respite care, transition care

## Abstract

**Objective:**

To evaluate the access of approved aged care services and factors associated with accessing these services.

**Methods:**

A retrospective cohort study was conducted (1/7/2003‐30/6/2013). The incidence of accessing permanent residential, home and respite care services within one year or transition care within 28 days of approval was evaluated. The association of participants’ socio‐demographic characteristics, limitations, health conditions and assessment characteristics with service use was evaluated.

**Results:**

In 799 750 older Australians, the incidence of accessing approved permanent residential care within one year was 70.9% (95% confidence interval [CI] 70.8%‐71.0%), home care 49.5% (95% CI 49.3%‐49.7%) and respite 41.8% (95% CI 41.7%‐41.9%). The incidence of accessing transition care at 28 days was 78.5% (95% CI 78.2%‐78.7%). Aged care seekers’, assessments’ and assessors’ characteristics are associated with service access.

**Conclusion:**

Monitoring the use of aged care service approvals is necessary for the identification of service access barriers to support evidence‐based policy changes.

**Policy Impact**

This study evaluated the access of aged care services in the Australian population. Monitoring the use of aged care services approvals is necessary for the identification of service access barriers to support evidence‐based policy changes.

**Practice Impact**

This study found that aged care seekers, assessment, and assessor characteristics were associated with people accessing services and may be considered barriers to services. This information is a first step to characterise the people still in need of care and potential barriers to aged care access.

## 1 INTRODUCTION

Australia has an ageing population, putting significant stress on its aged care and health‐care systems.^1^ Over 10% of Australians over 65 years old use government‐subsidised aged care services every year, costing the government $18 billion/year.^2^ These subsidies are granted after an aged care eligibility assessment in which the needs of older people are reviewed. In the 2017/2018 financial year, 186 128 new eligibility assessments were performed and historically most (83%) result in service approvals.^2^, ^3^ However, according to the most recent estimates (2009/2010) only 75% of individuals with approvals accessed these services.^3^

While there is significant literature on factors that lead to use of long‐term care,^4^, ^5^, ^6^, ^7^, ^8^, ^9^ less is understood about factors that lead to use of home, respite and transition care. Even less is understood about individuals that obtained approvals for support and did not access these services. Understanding the patterns of service access and characteristics of people accessing, or not accessing, these services can be useful to identify groups that may potentially experience barriers to entry into care or possibly groups where access is less prioritised. Additionally, a historical understanding of the access of these services is important for appropriate resource allocation and as a base for comparison for recent changes in the aged care sector (eg, Living Longer Living Better Act).^10^

This study's aims were to (a) characterise individuals with a first‐time aged care eligibility assessment and approvals for permanent residential, home, respite and transition care services and (b) investigate the factors associated with accessing an approved service.

## 2 METHODS

A retrospective cohort study was conducted using data from the Historical National Cohort of the Registry of Senior Australians (ROSA).^11^, ^12^ This data set is comprised of linked de‐identified data obtained from the Australian Institute of Health and Welfare’s National Aged Care Data Clearinghouse.^13^, ^14^, ^15^ In brief, these data sets contain information on aged care services eligibility assessments performed in Australia, the aged care seekers’ socio‐demographic characteristics, activity limitations, health problems, living conditions, carer availability, assessors, approvals for service, service utilisation and mortality.

The study cohort includes individuals ≥65 years old, not identified as being Aboriginal or Torres Strait Islander, who had a first‐time aged care eligibility assessment by an Aged Care Assessment Team (ACAT), and was approved for permanent residential, home, respite or transition care between 1/7/2003 and 30/6/2013 (N = 799 750). Individuals not approved for services were not included.

The cumulative incidence of accessing approved permanent residential, home or respite care within one year or receiving transition care within 28 days of approval was the outcome of interest. The cohort was followed until 30/6/2014 for one‐year follow‐up. One‐year follow‐up was chosen for permanent residential, home and respite care because the median time for service access ranges from 6 to 136 days, prior to 2009, the approvals were valid for one year,^3^ and this is generally agreed to be a long wait for services. Twenty‐eight days was used for transition care as approvals expire after this period.^14^

Factors associated with access to service evaluated included: age, sex, country of birth, Department of Veterans’ Affairs (DVA) Card Status,^16^ living arrangements, usual accommodation, carer availability, remoteness location, state, activity limitations and health conditions (Table 1). Assessor/assessment variables evaluated were as follows: assessors’ professional training, year, other approved services, priority category and emergency care.

**Table 1:** Characteristics of older Australians and their aged care assessments, by service approval group and status of accessing approved services within a year for permanent, home and respite care, and within 28 d for transition care, 2003‐2013

Variables	Categories	Permanent care approved (N = 656 263)		Home care approved (N = 397 419)		Respite care approved (N = 639 663)		Transition care approved^a^ (N = 100 738)	
		Care accessed, N (%)		Care accessed, N (%)		Care accessed, N (%)		Care accessed, N (%)	
		No	Yes	No	Yes	No	Yes	No	Yes
Total N		257 840 (39.3)	398 423 (60.7)	232 266 (58.4)	165 153 (41.6)	425 415 (66.5)	214 248 (33.5)	22 574 (22.4)	78 164 (77.6)
Person characteristics									
Age in y, median (IQR)		83 (78, 88)	84 (79, 89)	83 (78, 87)	82 (77, 87)	83 (78, 88)	84 (79, 88)	84 (78, 88)	82 (77, 87)
Sex	Female	150 234 (58.3)	246 887 (62.0)	138 832 (59.8)	107 528 (65.1)	257 737 (60.6)	132 853 (62.0)	13 338 (59.1)	50 032 (64.0)
	Male	107 192 (41.6)	150 733 (37.8)	93 107 (40.1)	57 451 (34.8)	166 973 (39.2)	81 057 (37.8)	9218 (40.8)	28 062 (35.9)
	Missing	414 (0.2)	803 (0.2)	327 (0.1)	174 (0.1)	705 (0.2)	338 (0.2)	18 (0.1)	70 (0.1)
Country of birth	Australia	174 950 (67.9)	282 958 (71.0)	159 438 (68.6)	108 995 (66.0)	290 762 (68.3)	152 211 (71.0)	13 586 (60.2)	52 394 (67.0)
	Born overseas	81 601 (31.6)	115 326 (28.9)	72 519 (31.2)	56 136 (34.0)	133 514 (31.4)	61 980 (28.9)	8946 (39.6)	25 739 (32.9)
	Missing	1289 (0.5)	139 (<0.01)	309 (0.1)	22 (<0.01)	1139 (0.3)	57 (<0.01)	42 (0.2)	31 (<0.01)
Department of Veterans' Affairs	No card	213 436 (82.8)	321 523 (80.7)	192 966 (83.1)	145 002 (87.8)	354 272 (83.3)	171 783 (80.2)	19 453 (86.2)	69 934 (89.5)
	Gold card	35 667 (13.8)	62 095 (15.6)	31 169 (13.4)	14 635 (8.9)	56 461 (13.3)	33 774 (15.8)	2574 (11.4)	6571 (8.4)
	White card	3805 (1.5)	6005 (1.5)	3575 (1.5)	2112 (1.3)	5917 (1.4)	3676 (1.7)	262 (1.2)	764 (1.0)
	Other card	4932 (1.9)	8800 (2.2)	4556 (2.0)	3404 (2.1)	8765 (2.1)	5015 (2.3)	285 (1.3)	895 (1.1)
Living arrangements	Institution care	2660 (1.0)	8917 (2.2)	1357 (0.6)	523 (0.3)	8169 (1.9)	2092 (1.0)	1520 (6.7)	1375 (1.8)
	Lives alone	105 886 (41.1)	199 010 (49.9)	97 928 (42.2)	80 365 (48.7)	185 063 (43.5)	94 965 (44.3)	10 469 (46.4)	40 294 (51.6)
	Lives with family	141 249 (54.8)	176 883 (44.4)	125 909 (54.2)	80 173 (48.5)	219 406 (51.6)	110 657 (51.6)	10 024 (44.4)	34 782 (44.5)
	Lives with others	6317 (2.4)	11 122 (2.8)	5311 (2.3)	3159 (1.9)	10 048 (2.4)	5048 (2.4)	526 (2.3)	1586 (2.0)
	Missing	1728 (0.7)	2491 (0.6)	1761 (0.8)	933 (0.6)	2729 (0.6)	1486 (0.7)	35 (0.2)	127 (0.2)
Usual accommodation	Private (owned or rental)	222 522 (86.3)	324 440 (81.4)	202 370 (87.1)	142 753 (86.4)	360 438 (84.7)	184 117 (85.9)	18 277 (81.0)	68 097 (87.1)
	Hotel/boarding house/hospital	4575 (1.8)	11 066 (2.8)	3663 (1.6)	1880 (1.1)	7676 (1.8)	4375 (2.0)	304 (1.3)	946 (1.2)
	Retirement village	24 130 (9.4)	46 912 (11.8)	22 261 (9.6)	18 196 (11.0)	43 494 (10.2)	22 086 (10.3)	2002 (8.9)	6992 (8.9)
	Residential aged care	1510 (0.6)	4406 (1.1)	766 (0.3)	299 (0.2)	6494 (1.5)	891 (0.4)	1435 (6.4)	1206 (1.5)
	Short term temporary supported	3462 (1.3)	9849 (2.5)	1490 (0.6)	1301 (0.8)	5017 (1.2)	1523 (0.7)	496 (2.2)	844 (1.1)
	Missing	1641 (0.6)	1750 (0.4)	1716 (0.7)	724 (0.4)	2296 (0.5)	1256 (0.6)	60 (0.3)	79 (0.1)
Carer availability	Has carer	218 484 (84.7)	324 513 (81.4)	198 528 (85.5)	135 365 (82.0)	355 361 (83.5)	184 888 (86.3)	17 269 (76.5)	59 951 (76.7)
	Has no carer	34 037 (13.2)	59 296 (14.9)	30 234 (13.0)	27 982 (16.9)	57 603 (13.5)	24 904 (11.6)	3687 (16.3)	16 284 (20.8)
	Not applicable	3459 (1.3)	11 434 (2.9)	1802 (0.8)	830 (0.5)	9474 (2.2)	2774 (1.3)	1576 (7.0)	1575 (2.0)
	Missing	1860 (0.7)	3180 (0.8)	1702 (0.7)	976 (0.6)	2977 (0.7)	1682 (0.8)	42 (0.2)	354 (0.5)
Remoteness	Major City	170 544 (66.1)	273 572 (68.7)	153 362 (66.0)	112 766 (68.3)	289 084 (68.0)	137 681 (64.3)	18 756 (83.1)	54 687 (70.0)
	Other	86 108 (33.4)	122 154 (30.7)	77 969 (33.6)	51 925 (31.4)	134 614 (31.6)	75 592 (35.3)	3643 (16.1)	22 782 (29.1)
	Missing	1188 (0.5)	2697 (0.7)	935 (0.4)	462 (0.3)	1717 (0.4)	975 (0.5)	175 (0.8)	695 (0.9)
State	ACT	5676 (2.2)	4178 (1.0)	5850 (2.5)	3175 (1.9)	7389 (1.7)	2945 (1.4)	404 (1.8)	1239 (1.6)
	NSW	86 813 (33.7)	137 952 (34.6)	96 461 (41.5)	55 148 (33.4)	143 135 (33.6)	89 900 (42.0)	2434 (10.8)	20 774 (26.6)
	NT	995 (0.4)	683 (0.2)	535 (0.2)	939 (0.6)	1158 (0.3)	596 (0.3)	1 (<0.01)	19 (<0.01)
	QLD	46 740 (18.1)	61 500 (15.4)	40 609 (17.5)	29 077 (17.6)	69 963 (16.4)	24 626 (11.5)	884 (3.9)	14 295 (18.3)
	SA	21 726 (8.4)	31 428 (7.9)	16 756 (7.2)	11 274 (6.8)	33 173 (7.8)	18 341 (8.6)	1833 (8.1)	7759 (9.9)
	TAS	5807 (2.3)	13 383 (3.4)	4497 (1.9)	4533 (2.7)	13 556 (3.2)	6870 (3.2)	168 (0.7)	2162 (2.8)
	VIC	61 938 (24.0)	114 830 (28.8)	38 317 (16.5)	40 923 (24.8)	111 825 (26.3)	55 335 (25.8)	11 158 (49.4)	24 697 (31.6)
	WA	28 145 (10.9)	34 469 (8.7)	29 241 (12.6)	20 084 (12.2)	45 216 (10.6)	15 635 (7.3)	5692 (25.2)	7219 (9.2)
Activity limitations^b^	Domestic assistance	244 883 (95.0)	363 330 (91.2)	224 000 (96.4)	160 696 (97.3)	399 530 (93.9)	203 337 (94.9)	19 537 (86.5)	72 230 (92.4)
	Transport	238 428 (92.5)	374 110 (93.9)	215 159 (92.6)	151 548 (91.8)	386 758 (90.9)	198 386 (92.6)	21 301 (94.4)	73 709 (94.3)
	Meals	226 334 (87.8)	351 371 (88.2)	204 538 (88.1)	141 627 (85.8)	362 765 (85.3)	191 097 (89.2)	19 003 (84.2)	68 960 (88.2)
	Social and community participation	223 142 (86.5)	353 934 (88.8)	200 171 (86.2)	142 387 (86.2)	362 605 (85.2)	185 431 (86.5)	19 504 (86.4)	66 066 (84.5)
	Health‐care tasks	211 547 (82.0)	351 130 (88.1)	183 104 (78.8)	124 229 (75.2)	338 347 (79.5)	176 672 (82.5)	20 382 (90.3)	66 772 (85.4)
	Home maintenance	200 776 (77.9)	287 341 (72.1)	181 905 (78.3)	129 175 (78.2)	323 084 (75.9)	160 542 (74.9)	16 193 (71.7)	57 271 (73.3)
	Self‐care	192 313 (74.6)	331 415 (83.2)	163 353 (70.3)	105 187 (63.7)	296 434 (69.7)	155 533 (72.6)	20 714 (91.8)	70 147 (89.7)
	Moving around places	168 851 (65.5)	278 096 (69.8)	143 756 (61.9)	90 814 (55.0)	255 842 (60.1)	128 786 (60.1)	17 666 (78.3)	60 810 (77.8)
	Movement activities	82 864 (32.1)	159 169 (39.9)	55 385 (23.8)	27 620 (16.7)	113 895 (26.8)	53 825 (25.1)	12 292 (54.5)	34 123 (43.7)
	Communication	55 685 (21.6)	102 246 (25.7)	44 861 (19.3)	26 624 (16.1)	79 555 (18.7)	43 621 (20.4)	4842 (21.4)	10 703 (13.7)
	Other	15 243 (5.9)	19 854 (5.0)	15 693 (6.8)	9928 (6.0)	22 953 (5.4)	14 203 (6.6)	424 (1.9)	2757 (3.5)
	None	521 (0.2)	774 (0.2)	365 (0.2)	163 (0.1)	936 (0.2)	365 (0.2)	75 (0.3)	286 (0.4)
Health conditions	Heart diseases	115 674 (44.9)	168 319 (42.2)	99 319 (42.8)	66 304 (40.1)	184 190 (43.3)	87 837 (41.0)	10 782 (47.8)	37 139 (47.5)
	Hypertension (high blood pressure)	112 921 (43.8)	171 356 (43.0)	104 319 (44.9)	75 851 (45.9)	191 644 (45.0)	92 480 (43.2)	10 733 (47.5)	40 623 (52.0)
	Arthritis	99 066 (38.4)	140 789 (35.3)	92 593 (39.9)	70 351 (42.6)	169 188 (39.8)	82 067 (38.3)	7071 (31.3)	28 491 (36.5)
	Diseases of the eye	65 378 (25.4)	93 410 (23.4)	59 639 (25.7)	43 884 (26.6)	109 157 (25.7)	54 355 (25.4)	4064 (18.0)	15 164 (19.4)
	History of cancer	62 152 (24.1)	73 723 (18.5)	46 086 (19.8)	26 621 (16.1)	88 412 (20.8)	37 867 (17.7)	4750 (21.0)	14 083 (18.0)
	Dementia	60 152 (23.3)	139 273 (35.0)	58 670 (25.3)	39 341 (23.8)	101 725 (23.9)	66 875 (31.2)	6074 (26.9)	11 023 (14.1)
	Diabetes	52 403 (20.3)	73 069 (18.3)	46 906 (20.2)	33 113 (20.0)	85 179 (20.0)	38 984 (18.2)	5149 (22.8)	17 621 (22.5)
	Cerebrovascular diseases	49 961 (19.4)	88 723 (22.3)	43 943 (18.9)	29 761 (18.0)	81 498 (19.2)	41 333 (19.3)	5816 (25.8)	19 857 (25.4)
	Chronic lower respiratory diseases	46 992 (18.2)	58 838 (14.8)	39 364 (16.9)	26 909 (16.3)	73 414 (17.3)	32 018 (14.9)	3969 (17.6)	13 573 (17.4)
	Osteoporosis	44 767 (17.4)	68 066 (17.1)	41 353 (17.8)	31 444 (19.0)	74 741 (17.6)	37 845 (17.7)	3621 (16.0)	14 623 (18.7)
	History of falls	43 194 (16.8)	82 457 (20.7)	40 720 (17.5)	26 790 (16.2)	72 565 (17.1)	38 516 (18.0)	5366 (23.8)	18 327 (23.4)
	Deafness/hearing loss	42 482 (16.5)	62 728 (15.7)	38 388 (16.5)	26 198 (15.9)	69 242 (16.3)	36 240 (16.9)	2538 (11.2)	9107 (11.7)
	Depression	39 441 (15.3)	62 999 (15.8)	36 126 (15.6)	27 807 (16.8)	64 077 (15.1)	35 632 (16.6)	3493 (15.5)	11 280 (14.4)
	Incontinence	38 317 (14.9)	71 243 (17.9)	30 089 (13)	19 491 (11.8)	55 590 (13.1)	29 900 (14.0)	3789 (16.8)	9845 (12.6)
	Pain	35 575 (13.8)	42 414 (10.6)	30 305 (13.0)	22 229 (13.5)	56 840 (13.4)	24 900 (11.6)	2155 (9.5)	9974 (12.8)
	Kidney and urinary system disorders	28 123 (10.9)	37 446 (9.4)	22 665 (9.8)	13 586 (8.2)	42 019 (9.9)	17 628 (8.2)	3025 (13.4)	9678 (12.4)
	Fracture	26 601 (10.3)	48 538 (12.2)	24 185 (10.4)	16 941 (10.3)	45 162 (10.6)	23 779 (11.1)	5139 (22.8)	25 592 (32.7)
	Bedsore	14 749 (5.7)	22 334 (5.6)	12 312 (5.3)	7819 (4.7)	22 927 (5.4)	10 864 (5.1)	1571 (7.0)	5274 (6.7)
	Malnutrition	6075 (2.4)	9791 (2.5)	5215 (2.2)	3463 (2.1)	9814 (2.3)	4813 (2.2)	688 (3.0)	2220 (2.8)
	Delirium	2551 (1.0)	7561 (1.9)	1906 (0.8)	967 (0.6)	4137 (1.0)	2283 (1.1)	893 (4.0)	2579 (3.3)
Assessment Characteristics									
Year	2003	2824 (1.1)	6582 (1.7)	2442 (1.1)	1965 (1.2)	5266 (1.2)	3290 (1.5)	‐	‐
	2004	10 513 (4.1)	22 595 (5.7)	10 206 (4.4)	6750 (4.1)	19 109 (4.5)	11 812 (5.5)	‐	‐
	2005	13 368 (5.2)	28 690 (7.2)	13 189 (5.7)	9477 (5.7)	24 133 (5.7)	15 583 (7.3)	25 (0.1)	4 (<0.01)
	2006	19 175 (7.4)	40 343 (10.1)	17 975 (7.7)	15 378 (9.3)	35 113 (8.3)	21 553 (10.1)	729 (3.2)	1590 (2.0)
	2007	22 210 (8.6)	43 738 (11.0)	20 597 (8.9)	18 005 (10.9)	38 982 (9.2)	22 870 (10.7)	2011 (8.9)	5799 (7.4)
	2008	27 095 (10.5)	45 780 (11.5)	23 952 (10.3)	18 624 (11.3)	44 800 (10.5)	25 325 (11.8)	2363 (10.5)	8016 (10.3)
	2009	30 257 (11.7)	45 895 (11.5)	26 449 (11.4)	19 295 (11.7)	49 185 (11.6)	25 757 (12.0)	2700 (12.0)	9523 (12.2)
	2010	28 826 (11.2)	46 049 (11.6)	26 315 (11.3)	19 412 (11.8)	49 184 (11.6)	25 248 (11.8)	3980 (17.6)	11 703 (15.0)
	2011	35 345 (13.7)	46 193 (11.6)	30 916 (13.3)	22 841 (13.8)	56 585 (13.3)	24 733 (11.5)	4134 (18.3)	15 494 (19.8)
	2012	42 913 (16.6)	47 787 (12.0)	37 580 (16.2)	22 483 (13.6)	65 774 (15.5)	25 028 (11.7)	4388 (19.4)	17 228 (22.0)
	2013	25 314 (9.8)	24 771 (6.2)	22 645 (9.7)	10 923 (6.6)	37 284 (8.8)	13 049 (6.1)	2244 (9.9)	8807 (11.3)
Assessors’ professional background^b^	Medical	121 612 (47.2)	223 048 (56.0)	111 180 (47.9)	66 485 (40.3)	196 815 (46.3)	95 639 (44.6)	19 528 (86.5)	63 098 (80.7)
	Nursing	198 300 (76.9)	326 082 (81.8)	175 305 (75.5)	121 130 (73.3)	324 418 (76.3)	164 924 (77.0)	20 842 (92.3)	72 765 (93.1)
	Health	126 470 (49.0)	224 788 (56.4)	108 098 (46.5)	74 032 (44.8)	210 512 (49.5)	99 191 (46.3)	19 541 (86.6)	68 673 (87.9)
	Social welfare	126 234 (49.0)	221 683 (55.6)	110 323 (47.5)	78 206 (47.4)	211 941 (49.8)	99 869 (46.6)	18 269 (80.9)	53 073 (67.9)
Service approvals	Home care	112 685 (43.7)	309 380 (77.7)	232 266 (100)	165 153 (100)	211 601 (49.7)	119 499 (55.8)	17 033 (75.5)	60 632 (77.6)
	Residential care (Permanent)	257 840 (100)	398 423 (100)	165 903 (71.4)	89 169 (54.0)	110 253 (25.9)	48 179 (22.5)	5989 (26.5)	52 866 (67.6)
	Respite care	228 624 (88.7)	262 511 (65.9)	209 114 (90.0)	135 707 (82.2)	425 415 (100)	214 248 (100)	13 523 (59.9)	56 515 (72.3)
	Transition care	12 421 (4.8)	23 611 (5.9)	13 792 (5.9)	5617 (3.4)	20 190 (4.7)	4077 (1.9)	22 574 (100)	78 164 (100)
	Emergency care	625 (0.2)	1458 (0.4)	595 (0.3)	215 (0.1)	436 (0.1)	1764 (0.8)	13 (0.1)	24 (<0.01)
Priority	Between 3 and 14 d	125 453 (48.7)	255 128 (64.0)	109 206 (47.0)	72 586 (44.0)	196 562 (46.2)	109 020 (50.9)	19 229 (85.2)	68 829 (88.1)
	More than 14 d	119 412 (46.3)	110 931 (27.8)	111 775 (48.1)	86 361 (52.3)	207 315 (48.7)	90 189 (42.1)	875 (3.9)	1526 (2.0)
	Within 48 h	12 618 (4.9)	31 529 (7.9)	10 884 (4.7)	5911 (3.6)	20 763 (4.9)	14 585 (6.8)	2456 (10.9)	7776 (9.9)
	Missing	357 (0.1)	835 (0.2)	401 (0.2)	295 (0.2)	775 (0.2)	454 (0.2)	14 (0.1)	33 (<0.01)

Note

IQR, Interquartile range.

a Transition care was established 2004‐2005, therefore no approvals for 2004 and 2005 are available. Transition care was evaluated within 28 d (and not one year as for other approvals).

b Missing data: assessors’ professional background (<1.1% for any cell), activity limitation (<0.2% for any cell)

Individuals were grouped by approval types for analysis, and the four types of approvals (non‐exclusive) are permanent residential, home, respite and transition care. To identify factors associated with accessing an approved service within the specified time period, Fine‐Gray subdistribution hazard regression models that accounted for the competing risk of death were employed. Time to accessing services was the difference between the dates of approval and entry to services. Cases were censored if they entered another service or reached the end of the study period. A stepwise variable selection approach was used, and Akaike information criterion was used for model selection. Only, cases with complete information were analysed (<3.4% of cases had missing data). Survival estimates, cumulative incidence function plots, and adjusted subdistribution hazard ratios (asHR) and 95% confidence intervals (CI) are provided. All tests were two‐sided and alpha = 0.0125 was considered statistically significant, which accounts for multiple hypothesis testing. R version 3.5.1 was used for analysis.

As a sensitivity analysis, for comparison to the estimates of a model treating death as a competing risk, a Cox regression model censoring individuals at the time of death was performed (Table S1).

## 3 RESULTS

Of the 799 750 individuals evaluated, 60.7% (N = 485 536) were female, 69.1% (N = 552 474) were born in Australia, 67.3% (N = 538 390) lived in major cities, and their median age was 83 years old (IQR: 78‐88). 82.1% (N = 656 263) were approved for permanent residential care, 49.7% (N = 397 419) for home care, 80.0% (N = 639 663) for respite care and 12.6% (N = 100 738) for transition care (Table 1).

**Figure 1:**
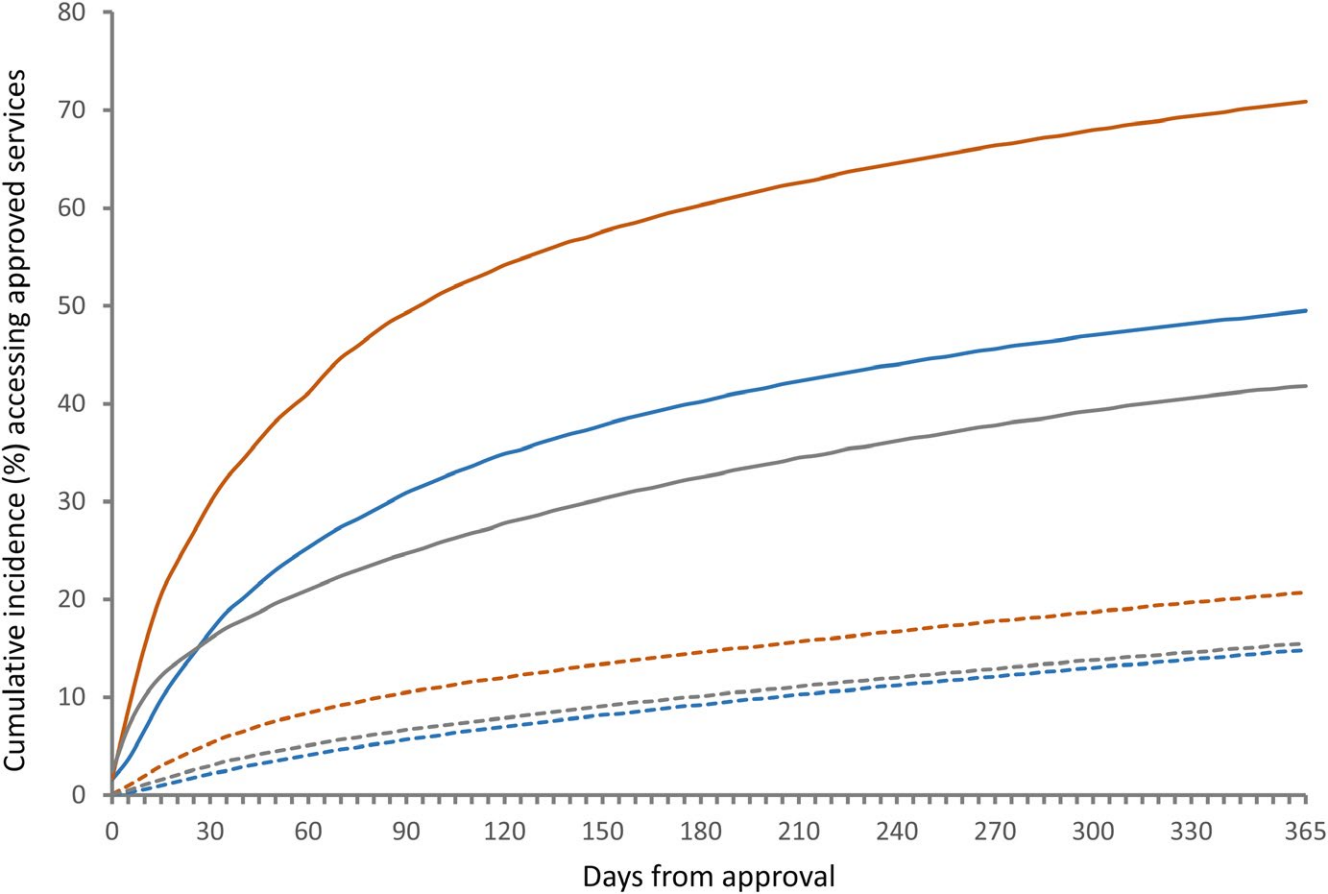
Cumulative incidence of accessing approved permanent residential, home and respite aged care services and incidence of death within one year. 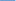 Home care; 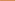 Permanent residential aged care; 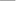 Respite care; 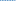 Death; 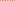 Death; 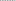 Death

**Figure 2:**
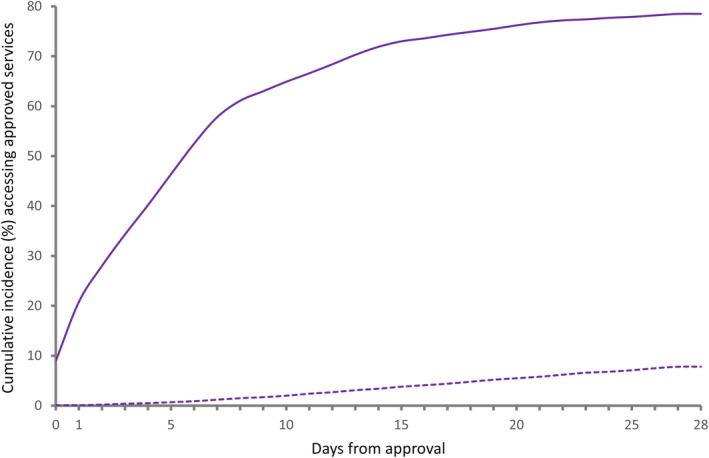
Cumulative incidence of accessing approved transition care and incidence of death within 28 days. 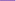 Transition care; 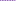 Death

Of 781 765 individuals with approvals for permanent residential, home or respite care, 221 131 (28.3%) did not access any services within one year, including 19 222 (2.5%) who died within that period before accessing services. Out of the 100 738 individuals with approvals for transition care, 22 574 (22.4%) did not access approved care within 28 days, including 2626 (2.7%) who died within that period. The cumulative incidence of accessing permanent residential care at one year was 70.9% (95% CI 70.8%‐71.0%), home care 49.5% (95% CI 49.3%‐49.7%) and respite care 41.8% (95% CI 41.7%‐41.9%) (Table 2, Figure 1). The cumulative incidence of accessing transition care at 28 days was 78.5% (95% CI 78.2%‐78.7%) (Table 2, Figure 2).

**Table 2:** Cumulative incidence of accessing aged care services after first aged care eligibility assessment approval^a^

Time (d)	Permanent care (N = 656 263)		Home care (N = 397 419)		Respite care (N = 639 663)		Time (d)	Transition care^b^ (N = 100 738)	
	N	Incidence %, (95%CI)	N	Incidence %, (95%CI)	N	Incidence %, (95%CI)		N	Incidence %, (95%CI)
30	416 706	29.9 (29.8, 30.0)	311 749	16.7 (16.6, 16.8)	466 057	16.0 (15.9, 16.1)	1	91 557	20.8 (20.6, 21.1)
90	267 382	49.3 (49.2, 49.5)	224 326	30.9 (30.7, 31.0)	365 264	24.7 (24.6, 24.8)	7	47 326	57.8 (57.5, 58.1)
180	188 824	60.3 (60.2, 60.4)	164 395	40.2 (40.1, 40.4)	288 179	32.5 (32.4, 32.6)	14	28 944	71.9 (71.6, 72.2)
365	118 863	70.9 (70.8, 71.0)	107 687	49.5 (49.3, 49.7)	204 395	41.8 (41.7, 41.9)	28	19 860	78.5 (78.2, 78.7)

Note

Abbreviations: CI, Confidence intervals.

a Censoring for entry into home care was performed for permanent residential care estimate. Censoring for entry into permanent residential care was performed for home care estimate.

b Transition care access was only evaluated for 28 d as these approvals are no longer valid after this period.^14^

### 3.1 Use of permanent residential aged care

Individuals using permanent residential care approvals were *less likely to (asHR, 95% CI):* be born overseas (0.92, 0.91‐0.92); live in temporary housing (0.95, 0.93‐0.97) or another residential care place (0.86, 0.81‐0.91); live outside of major cities (0.95, 0.94‐0.96); have limitations with domestic assistance (0.82, 0.80‐0.83), home maintenance (0.96, 0.95‐0.96); and have kidney and urinary system disorders (0.92, 0.90‐0.93), pain (0.93, 0.92‐0.95), cancer (0.81, 0.80‐0.82) and heart disease (0.96, 0.96‐0.97). People with other service approvals and priorities > 48 hours were less likely to access services (Table 3).

**Table 3:** Factors associated with accessing approved aged care services within one year for permanent, home, respite care, and within 28 d for transition care

Variables	Categories	Permanent care^a^ Adjusted Subdistribution HR (95%CI), N = 634 677	Home care^b^ Adjusted Subdistribution HR (95%CI), N = 386 469	Respite care^c^ Adjusted Subdistribution HR (95%CI), N = 620 425	Transition care^d^ AdjustedSubdistribution HR (95%CI), N = 98 859
Person Characteristics					
Age, y	Per 10‐y increments	1.11 (1.10, 1.11)	0.97 (0.96, 0.98)	1.09 (1.08, 1.09)	1.00 (0.99, 1.01)
Sex	Male vs Female	0.96 (0.95, 0.96)	0.93 (0.92, 0.94)	0.97 (0.96, 0.98)	0.98 (0.96, 0.99)
Country of birth	Born overseas vs Australia	0.92 (0.91, 0.92)	0.99 (0.98, 1.00)	0.94 (0.93, 0.95)	0.98 (0.96, 0.99)
Department of Veterans' Affairs card status	Gold vs No card	0.95 (0.94, 0.96)	0.72 (0.70, 0.73)	1.09 (1.08, 1.11)	0.89 (0.87, 0.92)
	White vs No card	1.04 (1.01, 1.06)	0.90 (0.86, 0.94)	1.17 (1.13, 1.21)	0.93 (0.87, 1.00)
	Other vs No card	1.03 (1.01, 1.06)	1.00 (0.97, 1.04)	1.04 (1.01, 1.07)	1.02 (0.95, 1.08)
Living arrangements	Institution care vs Lives alone	0.90 (0.85, 0.94)	0.35 (0.31, 0.39)	1.24 (1.14, 1.34)	0.97 (0.80, 1.17)
	Lives with family vs Lives alone	0.80 (0.79, 0.80)	0.85 (0.84, 0.86)	0.87 (0.86, 0.87)	1.00 (0.98, 1.01)
	Lives with others vs Lives alone	0.90 (0.88, 0.92)	0.84 (0.81, 0.87)	0.91 (0.89, 0.94)	0.99 (0.94, 1.04)
Usual accommodation	Hotel/boarding house/Hospital vs Private	1.09 (1.07, 1.12)	0.92 (0.87, 0.96)	1.02 (0.98, 1.06)	0.98 (0.92, 1.05)
	Temporary supported vs Private	0.95 (0.93, 0.97)	1.25 (1.18, 1.32)	0.68 (0.65, 0.72)	0.88 (0.82, 0.95)
	Residential aged care vs Private	0.86 (0.81, 0.91)	2.32 (1.93, 2.80)	0.12 (0.11, 0.13)	0.79 (0.67, 0.94)
	Retirement village vs Private	1.20 (1.19, 1.21)	1.20 (1.18, 1.22)	1.08 (1.07, 1.10)	0.98 (0.96, 1.00)
Carer availability	Yes vs No	1.00 (1.01, 0.99)	0.88 (0.87, 0.90)	1.06 (1.09, 1.05)	1.00 (0.98, 1.02)
Remoteness	Other vs Major city	0.95 (0.94, 0.96)	0.94 (0.93, 0.95)	1.14 (1.13, 1.15)	1.15 (1.13, 1.17)
State	ACT vs SA	0.97 (0.94, 1.01)	0.95 (0.91, 0.99)*	0.88 (0.84, 0.92)	1.26 (1.17, 1.34)
	NSW vs SA	1.06 (1.05, 1.08)	0.79 (0.78, 0.81)	1.13 (1.11, 1.15)	1.60 (1.56, 1.64)
	NT vs SA	0.62 (0.58, 0.67)	1.82 (1.69, 1.95)	0.59 (0.55, 0.65)	1.48 (1.01, 2.18)*
	QLD vs SA	0.94 (0.93, 0.96)	1.08 (1.06, 1.11)	0.68 (0.66, 0.69)	1.86 (1.81, 1.90)
	TAS vs SA	1.06 (1.03, 1.08)	1.12 (1.08, 1.16)	0.80 (0.78, 0.82)	2.07 (1.96, 2.19)
	VIC vs SA	0.97 (0.95, 0.98)	1.17 (1.14, 1.20)	0.90 (0.89, 0.92)	1.13 (1.10, 1.15)
	WA vs SA	0.97 (0.95, 0.99)	1.18 (1.15, 1.21)	0.71 (0.69, 0.72)	0.83 (0.81, 0.86)
Activity limitations					
Domestic assistance		0.82 (0.80, 0.83)	1.21 (1.17, 1.25)	0.84 (0.82, 0.86)	1.09 (1.05, 1.14)
Transport		1.05 (1.03, 1.06)	1.04 (1.02, 1.07)	1.11 (1.09, 1.13)	1.01 (0.98, 1.04)
Meals		1.25 (1.24, 1.27)	1.06 (1.04, 1.07)	1.28 (1.26, 1.30)	1.00 (0.97, 1.03)
Communication		1.03 (1.02, 1.04)	0.98 (0.97, 1.00)*	1.02 (1.01, 1.03)	0.93 (0.91, 0.95)
Social and community participation		1.11 (1.09, 1.12)	1.09 (1.07, 1.11)	1.09 (1.08, 1.11)	1.01 (0.99, 1.03)
Health‐care tasks		1.11 (1.09, 1.12)	1.01 (1.00, 1.02)	1.12 (1.11, 1.14)	1.00 (0.98, 1.02)
Home maintenance		0.96 (0.95, 0.96)	0.99 (0.98, 1.00)	0.95 (0.94, 0.96)	0.95 (0.94, 0.97)
Self‐care		1.23 (1.22, 1.25)	1.01 (0.99, 1.02)	1.23 (1.21, 1.24)	0.98 (0.95, 1.00)
Moving around places		1.01 (1.00, 1.02)*	0.96 (0.94, 0.97)	0.98 (0.97, 0.99)	0.96 (0.94, 0.97)
Movement activities		1.04 (1.03, 1.05)	0.87 (0.85, 0.88)	0.98 (0.97, 1.00)	0.90 (0.88, 0.91)
Health conditions					
Heart diseases		0.96 (0.96, 0.97)	0.98 (0.97, 0.99)	0.96 (0.96, 0.97)	1.01 (1.00, 1.03)
Hypertension		1.03 (1.02, 1.04)	1.02 (1.01, 1.03)	0.98 (0.97, 0.99)	1.04 (1.03, 1.05)
Arthritis		1.01 (1.00, 1.01)*	1.04 (1.03, 1.05)	0.97 (0.96, 0.98)	1.04 (1.03, 1.06)
Diseases of the eye		1.00 (1.00, 1.01)	1.05 (1.04, 1.06)	0.98 (0.97, 0.99)	1.04 (1.02, 1.06)
History of cancer		0.81 (0.80, 0.82)	0.90 (0.89, 0.91)	0.87 (0.86, 0.89)	0.96 (0.94, 0.98)
Dementia		1.31 (1.30, 1.32)	1.09 (1.08, 1.11)	1.27 (1.26, 1.29)	0.97 (0.94, 0.99)
Diabetes		1.00 (0.99, 1.01)	0.97 (0.96, 0.98)	0.97 (0.96, 0.98)	0.99 (0.97, 1.01)
Osteoporosis		1.00 (0.99, 1.01)	1.02 (1.01, 1.04)	1.00 (0.99, 1.02)	1.01 (0.99, 1.03)
History of falls		1.14 (1.13, 1.15)	1.03 (1.02, 1.04)	1.09 (1.08, 1.11)	1.03 (1.01, 1.05)
Depression		1.05 (1.04, 1.06)	1.04 (1.02, 1.05)	1.14 (1.13, 1.15)	1.01 (0.99, 1.03)
Incontinence		1.07 (1.06, 1.08)	1.04 (1.02, 1.05)	1.05 (1.04, 1.06)	1.00 (0.98, 1.02)
Pain		0.93 (0.92, 0.95)	1.02 (1.00, 1.03)	0.94 (0.93, 0.95)	1.03 (1.01, 1.05)
Kidney and urinary system disorders		0.92 (0.90, 0.93)	0.93 (0.91, 0.95)	0.93 (0.91, 0.94)	0.98 (0.96, 1.00)
Fracture		1.01 (1.00, 1.03)	1.00 (0.99, 1.02)	1.10 (1.08, 1.11)	1.04 (1.03, 1.06)
Bedsore		0.99 (0.98, 1.01)	0.98 (0.95, 1.00)*	0.99 (0.97, 1.01)	0.97 (0.94, 0.99)*
Delirium		1.14 (1.11, 1.17)	1.07 (1.00, 1.14)*	1.21 (1.16, 1.27)	1.06 (1.02, 1.10)
Assessment Characteristics					
Year	2003 vs 2013	1.38 (1.34, 1.42)	1.52 (1.44, 1.60)	1.53 (1.47, 1.60)	—e
	2004 vs 2013	1.27 (1.24, 1.29)	1.28 (1.24, 1.33)	1.42 (1.38, 1.46)	—e
	2005 vs 2013	1.25 (1.23, 1.28)	1.41 (1.37, 1.45)	1.43 (1.40, 1.47)	0.17 (0.06, 0.44)
	2006 vs 2013	1.30 (1.28, 1.32)	1.68 (1.64, 1.73)	1.46 (1.43, 1.50)	1.06 (1.00, 1.13)*
	2007 vs 2013	1.25 (1.23, 1.28)	1.69 (1.65, 1.73)	1.43 (1.40, 1.46)	1.00 (0.96, 1.04)
	2008 vs 2013	1.18 (1.16, 1.20)	1.59 (1.55, 1.63)	1.34 (1.31, 1.37)	1.00 (0.97, 1.03)
	2009 vs 2013	1.15 (1.14, 1.17)	1.46 (1.43, 1.50)	1.26 (1.23, 1.29)	0.98 (0.95, 1.01)
	2010 vs 2013	1.20 (1.18, 1.22)	1.45 (1.41, 1.48)	1.29 (1.26, 1.32)	0.96 (0.93, 0.99)
	2011 vs 2013	1.13 (1.11, 1.15)	1.47 (1.44, 1.50)	1.16 (1.14, 1.19)	1.01 (0.98, 1.03)
	2012 vs 2013	1.06 (1.04, 1.08)	1.20 (1.17, 1.23)	1.06 (1.03, 1.08)	0.98 (0.96, 1.01)
Assessors’ professional background					
Medical	Yes vs No	1.07 (1.06, 1.08)	0.89 (0.88, 0.90)	1.02 (1.01, 1.03)	1.00 (0.98, 1.02)
Nursing	Yes vs No	1.07 (1.06, 1.08)	1.05 (1.03, 1.06)	1.05 (1.04, 1.06)	1.05 (1.02, 1.08)
Social welfare	Yes vs No	1.05 (1.04, 1.06)	1.02 (1.01, 1.03)	0.98 (0.97, 0.99)	0.94 (0.92, 0.95)
Service approvals					
Home care	Yes vs No	0.50 (0.49, 0.50)	—f	0.68 (0.67, 0.68)	1.11 (1.09, 1.13)
Permanent care	Yes vs No	—f	0.70 (0.69, 0.71)	1.30 (1.29, 1.32)	0.57 (0.56, 0.58)
Respite care	Yes vs No	0.51 (0.51, 0.52)	0.83 (0.82, 0.84)	—f	0.98 (0.96, 1.01)
Transition care	Yes vs No	0.79 (0.77, 0.80)	0.64 (0.62, 0.66)	0.41 (0.39, 0.42)	—f
Priority	3 −14 d vs within 48 h	0.94 (0.93, 0.95)	1.14 (1.10, 1.17)	0.75 (0.73, 0.76)	0.99 (0.96, 1.01)
	≥14 d vs within 48 h	0.64 (0.63, 0.65)	1.06 (1.03, 1.09)	0.53 (0.52, 0.54)	0.80 (0.76, 0.84)

a Model N = 634 677. N = 21 586/656 263 = 3.3% cases excluded from final model due to missing data.

b Model N = 386 469. N = 10 950/397 419 = 2.8% cases excluded from final model due to missing data.

c Model N = 620 425. N = 19 238/639 663 = 3.0% cases excluded from final model due to missing data.

d Model N = 98 859. N = 1879/100 738 = 1.9% cases excluded from final model due to missing data.

e Transition care was established in 2004‐2005.

f Group being examined, variable not relevant in this model.

* *P* value between .0125 and .05, not considered statistically significant after correction for multiple hypothesis testing. All other estimates where confidence intervals do not include 1 have a *P* < .0125.

Individuals who accessed permanent residential care were *more likely to (asHR, 95%CI)*: be older, with a 1.11 (1.10‐1.11) increase in risk per 10‐year increase in age; live in hotels/boarding houses/hospitals (1.09, 1.07‐1.12) or retirement villages (1.20, 1.19‐1.21) compared to private homes; have limitations with communication (1.03, 1.02‐1.04), health‐care tasks (1.11, 1.09‐1.12), meals (1.25, 1.24‐1.27), movement activities (1.04, 1.03‐1.05), self‐care (1.23, 1.22‐1.25), social and community participation (1.11, 1.09‐1.12), and transport (1.05, 1.03‐1.06). Individuals with history of falls (1.14, 1.13‐1.15), delirium (1.14, 1.11‐1.17), dementia (1.31, 1.30‐1.32) and incontinence (1.07, 1.06‐1.08) were more likely to access their approved service. People with approvals prior to 2013 were more likely to access care than those in 2013, as were those whose assessment team included a medical practitioner, nursing professional and/or social welfare professional (Table 3).

### 3.2 Use of home care

Individuals accessing home care approvals were *less likely to (asHR, 95%CI):* have a gold (0.72, 0.70‐0.73) or white (0.90, 0.86‐0.94) DVA card; live with family (0.85, 0.84‐0.86), or with others (0.84, 0.81‐0.87) compared to living alone; live in a hotel/boarding house/hospital (0.92, 0.87‐0.96) compared to a private home; have a carer (0.88, 0.87‐0.90); live outside of major cities (0.94, 0.93‐0.95); have limitations with movement activities (0.87, 0.85‐0.88), moving around places (0.96, 0.94‐0.97); and have additional approvals for other types of care. People with cancer (0.90, 0.89‐0.91), diabetes (0.97, 0.96‐0.98) or heart disease (0.98, 0.97‐0.99) were less likely to use their approvals, as were those assessed by a team that included a medical practitioner (0.89, 0.89‐0.90) (Table 3).

Individuals using their home care approvals were *more likely to (asHR, 95%CI)*: be younger (0.97, 0.96‐0.98); live in temporary housing (1.25, 1.18‐1.32), residential aged care (2.32, 1.93‐2.80) or an independent unit within a retirement village (1.19, 1.17‐1.21) compared to living alone; have limitations with domestic tasks (1.21, 1.17‐1.25), meals (1.06, 1.04‐1.07), social and community participation (1.09, 1.07‐1.11), and transport (1.04, 1.02‐1.07); have dementia (1.09, 1.08‐1.11), arthritis (1.04, 1.03‐1.05), depression (1.04, 1.02‐1.05), eye diseases (1.05, 1.04‐1.06) or incontinence (1.04, 1.02‐1.05); have been assessed by a team that included a nursing or social welfare professional; have assessments prior to 2013; and have lower priority approvals (Table 3).

### 3.3 Use of respite care

Most characteristics associated with permanent residential care access were similar to respite care access. The main differences were that *(asHR, 95%CI)*: those with gold (1.09, 1.08‐1.11) DVA cards were more likely to use service approvals; people outside of major cities were more likely (1.14, 1.13‐1.15); people with limitations with moving around (0.98, 0.97‐0.99), hypertension (0.98, 0.97‐0.999), arthritis (0.97, 0.96‐0.98), diabetes (0.97, 0.96‐0.98) and eye diseases (0.98, 0.97‐0.99) were less likely; finally, those with approvals from a team with a social welfare professional were less likely to use respite services, while those with additional approvals for permanent residential care (1.30, 1.29‐1.32) were more likely (Table 3).

### 3.4 Use of transition care

Individuals accessing approved transition care were *less likely* to *(asHR, 95%CI)*: be males (0.98, 0.96‐0.99); be born overseas (0.98, 0.96‐0.99); have a gold DVA card (0.89, 0.87‐0.92); live in temporary supported housing (0.88, 0.82‐0.95) or residential aged care (0.79, 0.67‐0.94) compared to a private home; have limitations with communication (0.93, 0.91‐0.95), home maintenance (0.95, 0.94‐0.97), movement activities (0.90, 0.88‐0.91) and moving around places (0.96, 0.94‐0.98); have cancer (0.96, 0.94‐0.97) or dementia (0.96, 0.94‐0.97); have been assessed by a team with a social welfare professional (0.94, 0.92‐0.95); and have permanent residential care approvals (0.58, 0.56‐0.58).

Individuals accessing approved transition care were *more likely to (asHR, 95%CI)*: live outside major cities (1.15, 1.13‐1.17); have domestic task limitations (1.09, 1.05‐1.14); living with hypertension (1.04, 1.03‐1.05), pain (1.03, 1.01‐1.05), falls (1.03, 1.01‐1.05), arthritis (1.04, 1.03‐1.06), delirium (1.06, 1.02‐1.10), eye diseases (1.04, 1.02‐1.06) and fractures (1.04, 1.03‐1.06); and have additional home care approvals (1.11, 1.09‐1.13) (see Table 3).

## 4 DISCUSSION

Twenty‐eight per cent of individuals with approvals for permanent residential, home or respite aged care services did not access services within one year, and 22% with approvals for transition care did not access this service within 28 days. The cumulative incidence of accessing services varied between 42% and 79%. The access of services depended on individuals’ age, sex, living arrangements, state, remoteness and their specific limitations and health conditions (ie, pain, falls, cancer, delirium, dementia, diabetes, depression, fractures and incontinence) as well as external factors, including the eligibility assessment team and other service approvals. Our study highlights common factors associated with the use of aged care services but also factors important for specific type of service access.

Use of respite (42%) and home (50%) care approvals were lower than for permanent (71%) and transition (79%) care. Our estimates of access are lower for transition care (89%) and higher for permanent residential (49%), respite (25%) and home care (42%) than 2009 estimates.^3^ Our analysis included individuals from a 10‐year period, censored individuals that received other services and treated mortality as a competing risk during the follow‐up period, therefore reflecting the cumulative incidence of people with approvals at one year (or 28 days for transition care) who had not accessed other services or died, which is different from evaluating crude service access.^3^ As expected, approvals for transition care were the most often used services following approval, as these services are designed to assist hospitalised individuals in need of short‐term care to recover. The second most used approval was for permanent residential care, which is in line with this being the more commonly used longer term residential care solution in Australia (>72 000 entries in 2016).^2^, ^17^ The less frequent access of respite approvals could reflect the reported attitudes and concerns of older people about accessing these services.^18^, ^19^, ^20^ Finally, although not examined here, the lower access of home care services could be influenced by difficulties in accessing services (eg limited availability).^21^

The likelihood of utilising approved permanent residential care services was significantly higher for individuals born in Australia, living alone, in houses that they did not own, in major cities, with activity limitations and with history of falls, delirium, dementia and incontinence. These findings confirm prior strong evidence that age, functional limitations, living in homes that were not their own and having dementia are associated with entering residential care.^8^ Other factors, including incontinence, living alone^7^, ^8^ have also been reported in prior literature but less consistently. Australian‐specific factors were identified, including being born in Australia, living in a city and state variation. These factors are consistent with several reports^22^ that entry into care varies by race and cultural background.^5^, ^7^, ^8^

Functional limitations, living with dementia, incontinence and a history of falls were also factors associated with an individuals’ use of home care approvals. Additionally, being younger, female, living with family or others or in institutions, having a carer and living with arthritis, depression and eye diseases were specifically associated with accessing home care services. However, having a history of cancer or diabetes, having approvals for all other services and being assessed by a team with a medical practitioner made people less likely to access home care. These findings highlight that while staying at home is reportedly more desirable by older Australians^23^ and increasingly prevalent,^17^ the individuals entering these services are different than those entering residential care. Some of these differences suggest that these are individuals with health conditions manageable at home and with more carer or family support.

Factors associated with the use of respite service approvals were similar to those for permanent residential care approvals. These similarities are due to the overlap in the population accessing these services.^5^, ^6^ However, we also identified factors associated with the use of respite approvals only, including having a DVA card, living outside major cities and having hypertension, arthritis, diabetes, eye diseases and fractures. The differences in the effect of certain diseases could be related to the general demanding nature of certain conditions (ie, fractures). Unlike in the other approval groups, those with DVA benefits were more likely to access respite services, a reflection of veteran's options regarding services for longer term and transition care, but lack of additional options for respite. Contrary to people with approvals for home care and permanent residential care, those with respite approvals were more likely to use them if they were from outside major cities.

Individuals accessing transition care were more likely to have pain, arthritis, falls, fractures, eye diseases and delirium and have been assessed by teams that included a nursing professional and having additional approvals for home care. However, this is a service that men, individuals born overseas, and those who have support, are less likely to access. They are also less likely to access this care if they had the assessment done by a team that included a welfare professional, be in South Australia compared to most states or have approvals for permanent residential care. The high incidence of accessing these services and the conditions that characterise this cohort are consistent with our understanding that these individuals are in need of co‐ordinated and supportive posthospital discharge care.^24^

This is an observational study that relied on linked existing data sources, limiting our ability to comment on factors reported to be associated with the aged care services use (eg personal preferences, policy and availability) not in our data sets. For example, the national target provision rate of subsided aged care places likely influences the use of services and clustering effects related to these geographical allocations could exist, but we were unable to account for these in this analysis.^2^ However, a comprehensive set of characteristics of a national cohort was examined and several factors associated with entry into approved services were identified. Our study focused only on aged care services that required an aged care eligibility assessment by an ACAT and therefore does not evaluate other aged care services provided by the government or carer/family. Additionally, we did not examine the association of hospitalisations before the use of the service approval, which is also likely associated with the use of the services, particularly with transition care. However, we do not feel that the use of other minor aged care and hospital services would change the associations between the factors and services evaluated. Another limitation of our study is some weak but statistically significant associations; therefore, the strengths of the associations reported should be considered. We focused on the access of services within one year of approval, which underestimates the overall accessing of some services. It is possible individuals had additional eligibility assessments before 2009 or accessed services with their longer term approvals after 2009 and these were not captured in this study.

The strengths of this study include the national capture of people who underwent an aged care eligibility assessment and the use of a systematic approach that relies on trained assessors to collect its data, therefore increasing the internal validity of the information collected. Additionally, the data collection process was implemented in 2003 and remained the same during the study period. Ours is a population‐based study, and our findings are generalisable to the entire Australian population seeking and accessing aged care. Our analysis also addressed the people that died or entered a different service to obtain accurate estimates of service use by the end of the period, which had not been previously done.

Twenty‐two to twenty‐eight per cent of individuals approved for aged care services did not access them, and the cumulative incidence of accessing the four services varied between 42% and 79%. Aged care seekers’, assessments’ and assessors’ characteristics were associated with people accessing services, and some may be considered barriers to services. For example, while being female and having a history of dementia, falls, depression and incontinence is ubiquitously associated with individuals accessing aged care services, older individuals, with less support and more functional limitations and health conditions, were more likely to use residential care. This suggests that while home care is increasingly desirable for older individuals it does not seem to be accessed by those with major functional limitations, highlighting potential unmet needs for individuals with higher care requirements who may wish to remain at home. This information is a first step to characterise the people still in need of care and potential barriers to aged care access.

## CONFLICTS OF INTEREST

Renuka Visvanathan is a Board Member of Resthaven Inc, a not‐for‐profit aged care community service associated with the Uniting Church in Australia but separately incorporated, financially independent and a charitable Public Benevolent Institution. Craig Whitehead is a Board Member of Helping Hand Aged Care. All other authors have no conflicts of interest to declare.

## AUTHOR CONTRIBUTIONS

The following authors made substantial contributions to conception and design: MCI, ATA, SCEB, CW, MC, RV, SW. Acquisition of data: MCI, SCEB, SW. Analysis and interpretation of data: MCI, ATA. The manuscript was drafted by MCI, and it was critically revised with input from ATA, CW, SCEB, MC, RV and SW.

## ETHICAL CLEARANCE STATEMENT

This study received ethical approval from the University of South Australia's Human Research Ethics Committee (ID: 200489).
