# Supplementary material for: Factors associated with accessing aged care services in Australia after approval for services: Findings from the historical cohort of the Registry of Senior Australians
Source: Australas J Ageing. 2020 Jan 23;39(3):e382–92. doi: 10.1111/ajag.12760 (PMC7687099; doi:10.1111/ajag.12760)
Supplement: Supplementary file 1 [file AJAG-39-e382-s001.docx]

**Supplementary Table 1. Factors associated with accessing approved aged care services within one year for permanent, home, respite care, and within twenty-eight days for transition care. Comparison of Cox-regression and Fine Gray regression model estimates.**

| Variables |  | Permanent Care^1^  Adjusted HR (95%CI) | | Home Care^2^  Adjusted HR (95%CI) | | Respite Care^3^  Adjusted HR (95%CI) | | Transition Care^4^  Adjusted HR (95%CI) | |
| --- | --- | --- | --- | --- | --- | --- | --- | --- | --- |
|  | N | 634677 | | 386469 | | 620425 | | 98859 | |
| Person Characteristics | Categories | Cox model: death censored | **Fine-Gray Model** | Cox model: death censored | **Fine-Gray Model** | Cox model: death censored | **Fine-Gray Model** | Cox model: death censored | **Fine-Gray**  **Model** |
| Age, years | Per 10-year increments | 1.12(1.11, 1.12) | 1.11(1.10, 1.11) | 0.94(0.93, 0.94) | 0.97(0.96, 0.98) | 1.11(1.10, 1.11) | 1.09(1.08, 1.09) | 0.90(0.89, 0.91) | 1.00(0.99, 1.01) |
| Sex | Male vs. Female | 0.99(0.98, 1.00) | 0.96(0.95, 0.96) | 0.95(0.94, 0.96) | 0.93(0.92, 0.94) | 0.99(0.98, 1.00) | 0.97(0.96, 0.98) | 0.98(0.96, 1.00) | 0.98(0.96, 0.99) |
| Country of birth | Born overseas vs. Australia | 0.90(0.89, 0.91) | 0.92(0.91, 0.92) | 0.99(0.98, 1.00) | 0.99(0.98, 1.00) | 0.93(0.92, 0.94) | 0.94(0.93, 0.95) | 0.98(0.96, 0.99) | 0.98(0.96, 0.99) |
| Department of Veterans' Affairs card status | Gold vs. No card | 0.95(0.94, 0.96) | 0.95(0.94, 0.96) | 0.71(0.70, 0.72) | 0.72(0.70, 0.73) | 1.09(1.07, 1.10) | 1.09(1.08, 1.11) | 0.89(0.87, 0.92) | 0.89(0.87, 0.92) |
|  | White vs. No card | 1.00(0.98, 1.03) | 1.04(1.01, 1.06) | 0.87(0.83, 0.91) | 0.90(0.86, 0.94) | 1.15(1.11, 1.19) | 1.17(1.13, 1.21) | 0.94(0.87, 1.01) | 0.93(0.87, 1.00) |
|  | Other vs. No card | 1.02(1.00, 1.04) | 1.03(1.01, 1.06) | 0.99(0.95, 1.02) | 1.00(0.97, 1.04) | 1.02(0.99, 1.05) | 1.04(1.01, 1.07) | 1.01(0.95, 1.08) | 1.02(0.95, 1.08) |
| Living arrangements | Institution care vs. Lives alone | 0.84(0.81, 0.88) | 0.90(0.85, 0.94) | 0.76(0.65, 0.88) | 0.35(0.31, 0.39) | 1.10(1.02, 1.18) | 1.24(1.14, 1.34) | 0.96(0.79, 1.17) | 0.97(0.80, 1.17) |
|  | Lives with family vs. Lives alone | 0.79(0.79, 0.80) | 0.80(0.79, 0.80) | 0.85(0.84, 0.86) | 0.85(0.84, 0.86) | 0.86(0.85, 0.87) | 0.87(0.86, 0.87) | 1.00(0.98, 1.02) | 1.00(0.98, 1.01) |
|  | Lives with others vs. Lives alone | 0.87(0.85, 0.89) | 0.90(0.88, 0.92) | 0.82(0.79, 0.85) | 0.84(0.81, 0.87) | 0.89(0.86, 0.91) | 0.91(0.89, 0.94) | 0.99(0.94, 1.05) | 0.99(0.94, 1.04) |
| Usual accommodation | Hotel/boarding house/Hospital vs. Private | 1.05(1.02, 1.07) | 1.09(1.07, 1.12) | 0.89(0.85, 0.93) | 0.92(0.87, 0.96) | 0.99(0.95, 1.02) | 1.02(0.98, 1.06) | 0.98(0.92, 1.05) | 0.98(0.92, 1.05) |
|  | Temporary supported vs. Private | 0.88(0.86, 0.09) | 0.95(0.93, 0.97) | 1.19(1.12, 1.25) | 1.25(1.18, 1.32) | 0.62(0.59, 0.66) | 0.68(0.65, 0.72) | 0.88(0.83, 0.95) | 0.88(0.82, 0.95) |
|  | Residential aged care vs. Private | 0.74(0.71, 0.78) | 0.86(0.81, 0.91) | 1.12(0.93, 1.35) | 2.32(1.93, 2.80) | 0.13(0.11, 0.14) | 0.12(0.11, 0.13) | 0.80(0.68, 0.95) | 0.79(0.67, 0.94) |
|  | Retirement village vs. Private | 1.20(1.19, 1.21) | 1.20(1.19, 1.21) | 1.19(1.17, 1.21) | 1.20(1.18, 1.22) | 1.07(1.06, 1.09) | 1.08(1.07, 1.10) | 0.98(0.96, 1.01) | 0.98(0.96, 1.00) |
| Carer availability | Yes vs. No | 1.03(1.02, 1.04) | 1.00(1.01, 0.99) | 0.89(0.88, 0.90) | 0.88(0.90, 0.87) | 1.09(1.07, 1.10) | 1.06(1.09, 1.05) | 1.00(0.98, 1.02) | 1.00(0.98, 1.02) |
| Remoteness | Other vs. Major city | 0.94(0.93, 0.95) | 0.95(0.94, 0.96) | 0.95(0.94, 0.96) | 0.94(0.93, 0.95) | 1.14(1.13, 1.15) | 1.14(1.13, 1.15) | 1.15(1.13, 1.17) | 1.15(1.13, 1.17) |
| State | ACT vs. SA | 0.93(0.89, 0.96) | 0.97(0.94, 1.01) | 0.95(0.91, 0.99) | 0.95(0.91, 0.99) | 0.83(0.80, 0.87) | 0.88(0.84, 0.92) | 1.26(1.18, 1.35) | 1.26(1.17, 1.34) |
|  | NSW vs. SA | 1.06(1.05, 1.07) | 1.06(1.05, 1.08) | 0.80(0.79, 0.82) | 0.79(0.78, 0.81) | 1.12(1.10, 1.14) | 1.13(1.11, 1.15) | 1.61(1.56, 1.65) | 1.60(1.56, 1.64) |
|  | NT vs. SA | 0.58(0.53, 0.62) | 0.62(0.58, 0.67) | 1.67(1.56, 1.79) | 1.82(1.69, 1.95) | 0.72(0.67, 0.78) | 0.59(0.55, 0.65) | 1.48(0.94, 2.32) | 1.48(1.01, 2.18) |
|  | QLD vs. SA | 0.92(0.91, 0.94) | 0.94(0.93, 0.96) | 1.08(1.06, 1.10) | 1.08(1.06, 1.11) | 0.66(0.65, 0.68) | 0.68(0.66, 0.69) | 1.86(1.81, 1.92) | 1.86(1.81, 1.90) |
|  | TAS vs. SA | 1.06(1.04, 1.08) | 1.06(1.03, 1.08) | 1.12(1.08, 1.16) | 1.12(1.08, 1.16) | 0.79(0.77, 0.82) | 0.80(0.78, 0.82) | 2.09(1.99, 2.20) | 2.07(1.96, 2.19) |
|  | VIC vs. SA | 0.96(0.94, 0.97) | 0.97(0.95, 0.98) | 1.18(1.15, 1.20) | 1.17(1.14, 1.20) | 0.89(0.88, 0.91) | 0.90(0.89, 0.92) | 1.13(1.10, 1.17) | 1.13(1.10, 1.15) |
|  | WA vs. SA | 0.97(0.96, 0.99) | 0.97(0.95, 0.99) | 1.20(1.17, 1.23) | 1.18(1.15, 1.21) | 0.71(0.70, 0.73) | 0.71(0.69, 0.72) | 0.84(0.81, 0.87) | 0.83(0.81, 0.86) |
| Activity limitation | |  |  |  |  |  |  |  |  |
| Communication | Yes vs. No | 1.04(1.03, 1.05) | 1.03(1.02, 1.04) | 0.98(0.97, 0.99) | 0.98(0.97, 1.00) | 1.02(1.00, 1.03) | 1.02(1.01, 1.03) | 0.93(0.91, 0.95) | 0.93(0.91, 0.95) |
| Domestic assistance | Yes vs. No | 0.80(0.78, 0.81) | 0.82(0.80, 0.83) | 1.22(1.18, 1.26) | 1.21(1.17, 1.25) | 0.83(0.81, 0.85) | 0.84(0.82, 0.86) | 1.09(1.05, 1.14) | 1.09(1.05, 1.14) |
| Health care tasks | Yes vs. No | 1.14(1.13, 1.16) | 1.11(1.09, 1.12) | 1.02(1.01, 1.04) | 1.01(1.00, 1.02) | 1.14(1.13, 1.16) | 1.12(1.11, 1.14) | 1.00(0.98, 1.02) | 1.00(0.98, 1.02) |
| Home maintenance | Yes vs. No | 0.96(0.95, 0.97) | 0.96(0.95, 0.96) | 0.99(0.98, 1.00) | 0.99(0.98, 1.00) | 0.95(0.94, 0.96) | 0.95(0.94, 0.96) | 0.95(0.94, 0.97) | 0.95(0.94, 0.97) |
| Meals | Yes vs. No | 1.27(1.25, 1.29) | 1.25(1.24, 1.27) | 1.06(1.05, 1.08) | 1.06(1.04, 1.07) | 1.29(1.27, 1.31) | 1.28(1.26, 1.30) | 1.00(0.97, 1.03) | 1.00(0.97, 1.03) |
| Movement activities | Yes vs. No | 1.17(1.16, 1.18) | 1.04(1.03, 1.05) | 0.90(0.89, 0.92) | 0.87(0.85, 0.88) | 1.05(1.04, 1.06) | 0.98(0.97, 1.00) | 0.90(0.89, 0.92) | 0.90(0.88, 0.91) |
| Self-care | Yes vs. No | 1.29(1.28, 1.31) | 1.23(1.22, 1.25) | 1.03(1.01, 1.04) | 1.01(0.99, 1.02) | 1.26(1.24, 1.27) | 1.23(1.21, 1.24) | 0.98(0.95, 1.00) | 0.98(0.95, 1.00) |
| Social and community participation | Yes vs. No | 1.10(1.09, 1.11) | 1.11(1.09, 1.12) | 1.08(1.07, 1.10) | 1.09(1.07, 1.11) | 1.09(1.07, 1.1) | 1.09(1.08, 1.11) | 1.01(0.99, 1.04) | 1.01(0.99, 1.03) |
| Transport | Yes vs. No | 1.06(1.05, 1.08) | 1.05(1.03, 1.06) | 1.05(1.03, 1.08) | 1.04(1.02, 1.07) | 1.12(1.10, 1.14) | 1.11(1.09, 1.13) | 1.01(0.98, 1.05) | 1.01(0.98, 1.04) |
| Moving around places at or away from home | Yes vs. No | 1.04(1.03, 1.05) | 1.01(1.00, 1.02) | 0.96(0.95, 0.97) | 0.96(0.94, 0.97) | 1.00(0.99, 1.01) | 0.98(0.97, 0.99) | 0.96(0.94, 0.98) | 0.96(0.94, 0.97) |
| Health conditions | |  |  |  |  |  |  |  |  |
| Hypertension | Yes vs. No | 1.01(1.00, 1.01) | 1.03(1.02, 1.04) | 1.01(1.00, 1.02) | 1.02(1.01, 1.03) | 0.97(0.96, 0.98) | 0.98(0.97, 0.99) | 1.04(1.02, 1.05) | 1.04(1.03, 1.05) |
| Osteoporosis | Yes vs. No | 0.98(0.98, 0.99) | 1.00(0.99, 1.01) | 1.02(1.00, 1.03) | 1.02(1.01, 1.04) | 0.99(0.98, 1.00) | 1.00(0.99, 1.02) | 1.01(0.99, 1.03) | 1.01(0.99, 1.03) |
| Kidney & urinary system disorders | Yes vs. No | 0.98(0.97, 0.99) | 0.92(0.90, 0.93) | 0.95(0.94, 0.97) | 0.93(0.91, 0.95) | 0.96(0.95, 0.98) | 0.93(0.91, 0.94) | 0.99(0.97, 1.01) | 0.98(0.96, 1.00) |
| Pain | Yes vs. No | 0.95(0.94, 0.96) | 0.93(0.92, 0.95) | 1.02(1.01, 1.04) | 1.02(1.00, 1.03) | 0.95(0.93, 0.96) | 0.94(0.93, 0.95) | 1.03(1.01, 1.05) | 1.03(1.01, 1.05) |
| Falls | Yes vs. No | 1.10(1.09, 1.11) | 1.14(1.13, 1.15) | 1.02(1.01, 1.03) | 1.03(1.02, 1.04) | 1.07(1.06, 1.09) | 1.09(1.08, 1.11) | 1.03(1.01, 1.04) | 1.03(1.01, 1.05) |
| Arthritis | Yes vs. No | 0.97(0.96, 0.98) | 1.01(1.00, 1.01) | 1.03(1.01, 1.04) | 1.04(1.03, 1.05) | 0.95(0.94, 0.96) | 0.97(0.96, 0.98) | 1.04(1.03, 1.06) | 1.04(1.03, 1.06) |
| Bedsore | Yes vs. No | 1.01(1.00, 1.03) | 0.99(0.98, 1.01) | 0.99(0.96, 1.01) | 0.98(0.95, 1.00) | 1.00(0.98, 1.02) | 0.99(0.97, 1.01) | 0.97(0.94, 1.00) | 0.97(0.94, 0.99) |
| Cancer | Yes vs. No | 0.98(0.98, 0.99) | 0.81(0.80, 0.82) | 0.95(0.94, 0.97) | 0.90(0.89, 0.91) | 0.97(0.95, 0.98) | 0.87(0.86, 0.89) | 0.97(0.95, 0.99) | 0.96(0.94, 0.98) |
| Delirium | Yes vs. No | 1.15(1.12, 1.17) | 1.14(1.11, 1.17) | 1.02(0.96, 1.09) | 1.07(1.00, 1.14) | 1.18(1.13, 1.23) | 1.21(1.16, 1.27) | 1.06(1.02, 1.10) | 1.06(1.02, 1.10) |
| Dementia | Yes vs. No | 1.21(1.20, 1.22) | 1.31(1.30, 1.32) | 1.06(1.05, 1.08) | 1.09(1.08, 1.11) | 1.22(1.20, 1.23) | 1.27(1.26, 1.29) | 0.96(0.94, 0.98) | 0.97(0.94, 0.99) |
| Depression | Yes vs. No | 1.01(1.00, 1.02) | 1.05(1.04, 1.06) | 1.03(1.01, 1.04) | 1.04(1.02, 1.05) | 1.11(1.10, 1.13) | 1.14(1.13, 1.15) | 1.01(0.99, 1.03) | 1.01(0.99, 1.03) |
| Diabetes | Yes vs. No | 0.98(0.97, 0.99) | 1.00(0.99, 1.01) | 0.97(0.96, 0.98) | 0.97(0.96, 0.98) | 0.96(0.95, 0.97) | 0.97(0.96, 0.98) | 0.99(0.97, 1.01) | 0.99(0.97, 1.01) |
| Diseases of the eye | Yes vs. No | 0.98(0.97, 0.99) | 1.00(1.00, 1.01) | 1.03(1.02, 1.05) | 1.05(1.04, 1.06) | 0.96(0.95, 0.97) | 0.98(0.97, 0.99) | 1.04(1.02, 1.06) | 1.04(1.02, 1.06) |
| Fracture | Yes vs. No | 0.96(0.95, 0.97) | 1.01(1.00, 1.03) | 0.98(0.97, 1.00) | 1.00(0.99, 1.02) | 1.07(1.05, 1.08) | 1.10(1.08, 1.11) | 1.04(1.02, 1.05) | 1.04(1.03, 1.06) |
| Heart | Yes vs. No | 1.00(0.99, 1.00) | 0.96(0.96, 0.97) | 0.99(0.98, 1.00) | 0.98(0.97, 0.99) | 0.98(0.97, 0.99) | 0.96(0.96, 0.97) | 1.02(1.00, 1.03) | 1.01(1.00, 1.03) |
| Incontinence | Yes vs. No | 1.07(1.06, 1.08) | 1.07(1.06, 1.08) | 1.03(1.02, 1.05) | 1.04(1.02, 1.05) | 1.04(1.03, 1.05) | 1.05(1.04, 1.06) | 1.00(0.98, 1.02) | 1.00(0.98, 1.02) |
| Assessment Characteristics | |  |  |  |  |  |  |  |  |
| Calendar year | 2003 vs. 2013 | 1.39(1.35, 1.43) | 1.38(1.34, 1.42) | 1.53(1.45, 1.61) | 1.52(1.44, 1.60) | 1.55(1.49, 1.62) | 1.53(1.47, 1.60) | -^5^ | -^5^ |
|  | 2004 vs. 2013 | 1.32(1.30, 1.35) | 1.27(1.24, 1.29) | 1.30(1.26, 1.35) | 1.28(1.24, 1.33) | 1.48(1.44, 1.52) | 1.42(1.38, 1.46) | -^5^ | -^5^ |
|  | 2005 vs. 2013 | 1.30(1.27, 1.32) | 1.25(1.23, 1.28) | 1.44(1.39, 1.48) | 1.41(1.37, 1.45) | 1.48(1.45, 1.52) | 1.43(1.40, 1.47) | 0.15(0.05, 0.48) | 0.17(0.06, 0.44) |
|  | 2006 vs. 2013 | 1.34(1.32, 1.37) | 1.30(1.28, 1.32) | 1.70(1.66, 1.74) | 1.68(1.64, 1.73) | 1.51(1.47, 1.54) | 1.46(1.43, 1.50) | 1.07(1.01, 1.13) | 1.06(1.00, 1.13) |
|  | 2007 vs. 2013 | 1.29(1.27, 1.32) | 1.25(1.23, 1.28) | 1.72(1.68, 1.76) | 1.69(1.65, 1.73) | 1.47(1.44, 1.50) | 1.43(1.40, 1.46) | 1.00(0.96, 1.03) | 1.00(0.96, 1.04) |
|  | 2008 vs. 2013 | 1.21(1.19, 1.23) | 1.18(1.16, 1.20) | 1.61(1.57, 1.65) | 1.59(1.55, 1.63) | 1.37(1.34, 1.40) | 1.34(1.31, 1.37) | 0.99(0.96, 1.03) | 1.00(0.97, 1.03) |
|  | 2009 vs. 2013 | 1.17(1.15, 1.18) | 1.15(1.14, 1.17) | 1.47(1.44, 1.51) | 1.46(1.43, 1.50) | 1.27(1.24, 1.30) | 1.26(1.23, 1.29) | 0.98(0.95, 1.01) | 0.98(0.95, 1.01) |
|  | 2010 vs. 2013 | 1.23(1.21, 1.25) | 1.20(1.18, 1.22) | 1.46(1.43, 1.50) | 1.45(1.41, 1.48) | 1.30(1.28, 1.33) | 1.29(1.26, 1.32) | 0.96(0.93, 0.99) | 0.96(0.93, 0.99) |
|  | 2011 vs. 2013 | 1.15(1.13, 1.17) | 1.13(1.11, 1.15) | 1.48(1.44, 1.51) | 1.47(1.44, 1.50) | 1.17(1.15, 1.20) | 1.16(1.14, 1.19) | 1.01(0.98, 1.03) | 1.01(0.98, 1.03) |
|  | 2012 vs. 2013 | 1.07(1.06, 1.09) | 1.06(1.04, 1.08) | 1.21(1.18, 1.23) | 1.20(1.17, 1.23) | 1.06(1.04, 1.09) | 1.06(1.03, 1.08) | 0.98(0.96, 1.01) | 0.98(0.96, 1.01) |
| Assessors’ professional background | |  |  |  |  |  |  |  |  |
| Medical practitioners | Yes vs. No | 1.09(1.08, 1.10) | 1.07(1.06, 1.08) | 0.90(0.89, 0.91) | 0.89(0.88, 0.90) | 1.03(1.02, 1.04) | 1.02(1.01, 1.03) | 1.00(0.98, 1.02) | 1.00(0.98, 1.02) |
| Nursing professionals | Yes vs. No | 1.10(1.09, 1.11) | 1.07(1.06, 1.08) | 1.05(1.04, 1.06) | 1.05(1.03, 1.06) | 1.06(1.05, 1.07) | 1.05(1.04, 1.06) | 1.05(1.02, 1.08) | 1.05(1.02, 1.08) |
| Social welfare professionals | Yes vs. No | 1.06(1.05, 1.06) | 1.05(1.04, 1.06) | 1.03(1.01, 1.04) | 1.02(1.01, 1.03) | 0.98(0.97, 0.99) | 0.98(0.97, 0.99) | 0.94(0.92, 0.95) | 0.94(0.92, 0.95) |
| Service approvals | |  |  |  |  |  |  |  |  |
| Home care | Yes vs. No | 0.47(0.47, 0.47) | 0.50(0.49, 0.50) | -^6^ | -^6^ | 0.66(0.65, 0.66) | 0.68(0.67, 0.68) | 1.11(1.08, 1.13) | 1.11(1.09, 1.13) |
| Permanent care | Yes vs. No | -^6^ | -^6^ | 0.71(0.70, 0.72) | 0.70(0.69, 0.71) | 1.34(1.33, 1.36) | 1.30(1.29, 1.32) | 0.58(0.57, 0.59) | 0.57(0.56, 0.58) |
| Respite care | Yes vs. No | 0.48(0.47, 0.48) | 0.51(0.51, 0.52) | 0.83(0.82, 0.84) | 0.83(0.82, 0.84) | -^6^ | -^6^ | 0.98(0.96, 1.00) | 0.98(0.96, 1.01) |
| Transition care | Yes vs. No | 0.77(0.76, 0.78) | 0.79(0.77, 0.80) | 0.64(0.62, 0.66) | 0.64(0.62, 0.66) | 0.39(0.38, 0.41) | 0.41(0.39, 0.42) | -^6^ | -^6^ |
| Priority | 3 -14 days vs. within 48 hours | 0.94(0.92, 0.95) | 0.94(0.93, 0.95) | 1.13(1.10, 1.16) | 1.14(1.10, 1.17) | 0.75(0.73, 0.76) | 0.75(0.73, 0.76) | 0.98(0.96, 1.01) | 0.99(0.96, 1.01) |
|  | ≥14 days vs. within 48 hours | 0.59(0.58, 0.60) | 0.64(0.63, 0.65) | 1.04(1.01, 1.06) | 1.06(1.03, 1.09) | 0.51(0.50, 0.52) | 0.53(0.52, 0.54) | 0.79(0.75, 0.84) | 0.80(0.76, 0.84) |

1. Model N=634677. N=21586/656263=3.3% cases excluded from final model due to missing data.
2. Model N=386469. N=10950/397419=2.8% cases excluded from final model due to missing data.
3. Model N=620425. N=19238/639663=3.0% cases excluded from final model due to missing data.
4. Model N=98859. N=1879/100738=1.9% cases excluded from final model due to missing data.
5. Transition care was established 2004-2005.
6. Group being examined, variable not relevant in this model.
