# Supplementary material for: Factors associated with accessing aged care services in Australia after approval for services: Findings from the historical cohort of the Registry of Senior Australians
Source: Australas J Ageing. 2020 Jan 23;39(3):e382–92. doi: 10.1111/ajag.12760 (PMC7687099; doi:10.1111/ajag.12760)
Supplement: Supplementary file 2 [file AJAG-39-e382-s002.docx]

**Supplementary Table 2. Characteristics of older Australians and their aged care assessments, by service approval group and status of accessing approved services within a year for permanent, home, and respite care, and within 28 days for transition care, 2003-2013. (Full version of Table 1 in main text.)**

| Variables | Categories | | Permanent Care Approved  (N=656263) | | | | Home Care Approved  (N=397419) | | | | Respite Care Approved (N=639663) | | | | Transition Care Approved ^1^ (N=100738) | | | |
| --- | --- | --- | --- | --- | --- | --- | --- | --- | --- | --- | --- | --- | --- | --- | --- | --- | --- | --- |
|  |  | | Care accessed, N (%) | | | | Care accessed, N (%) | | | | Care accessed, N (%) | | | | Care accessed, N (%) | | | |
|  |  | | No | | Yes | | No | | Yes | | No | | Yes | | No | | Yes | |
| Total N |  | | 257840(39.3) | | 398423(60.7) | | 232266(58.4) | | 165153(41.6) | | 425415(66.5) | | 214248(33.5) | | 22574(22.4) | | 78164(77.6) | |
| Person Characteristics | |  | |  | |  | |  | |  | |  | |  | |  | |  |
| Age in years, median (IQR) |  | | 83(78, 88) | | 84(79, 89) | | 83(78, 87) | | 82(77, 87) | | 83(78, 88) | | 84(79, 88) | | 84(78, 88) | | 82(77, 87) | |
| Sex | Female | | 150234(58.3) | | 246887(62.0) | | 138832(59.8) | | 107528(65.1) | | 257737(60.6) | | 132853(62.0) | | 13338(59.1) | | 50032(64.0) | |
|  | Male | | 107192(41.6) | | 150733(37.8) | | 93107(40.1) | | 57451(34.8) | | 166973(39.2) | | 81057(37.8) | | 9218(40.8) | | 28062(35.9) | |
|  | Missing | | 414(0.2) | | 803(0.2) | | 327(0.1) | | 174(0.1) | | 705(0.2) | | 338(0.2) | | 18(0.1) | | 70(0.1) | |
| Country of birth | Australia | | 174950(67.9) | | 282958(71.0) | | 159438(68.6) | | 108995(66.0) | | 290762(68.3) | | 152211(71.0) | | 13586(60.2) | | 52394(67.0) | |
|  | Born overseas | | 81601(31.6) | | 115326(28.9) | | 72519(31.2) | | 56136(34.0) | | 133514(31.4) | | 61980(28.9) | | 8946(39.6) | | 25739(32.9) | |
|  | Missing | | 1289(0.5) | | 139(<0.01) | | 309(0.1) | | 22(<0.01) | | 1139(0.3) | | 57(<0.01) | | 42(0.2) | | 31(<0.01) | |
| Department of Veterans' Affairs | No card | | 213436(82.8) | | 321523(80.7) | | 192966(83.1) | | 145002(87.8) | | 354272(83.3) | | 171783(80.2) | | 19453(86.2) | | 69934(89.5) | |
|  | Gold card | | 35667(13.8) | | 62095(15.6) | | 31169(13.4) | | 14635(8.9) | | 56461(13.3) | | 33774(15.8) | | 2574(11.4) | | 6571(8.4) | |
|  | White card | | 3805(1.5) | | 6005(1.5) | | 3575(1.5) | | 2112(1.3) | | 5917(1.4) | | 3676(1.7) | | 262(1.2) | | 764(1.0) | |
|  | Other card | | 4932(1.9) | | 8800(2.2) | | 4556(2.0) | | 3404(2.1) | | 8765(2.1) | | 5015(2.3) | | 285(1.3) | | 895(1.1) | |
| Living arrangements | Institution care | | 2660(1.0) | | 8917(2.2) | | 1357(0.6) | | 523(0.3) | | 8169(1.9) | | 2092(1.0) | | 1520(6.7) | | 1375(1.8) | |
|  | Lives alone | | 105886(41.1) | | 199010(49.9) | | 97928(42.2) | | 80365(48.7) | | 185063(43.5) | | 94965(44.3) | | 10469(46.4) | | 40294(51.6) | |
|  | Lives with family | | 141249(54.8) | | 176883(44.4) | | 125909(54.2) | | 80173(48.5) | | 219406(51.6) | | 110657(51.6) | | 10024(44.4) | | 34782(44.5) | |
|  | Lives with others | | 6317(2.4) | | 11122(2.8) | | 5311(2.3) | | 3159(1.9) | | 10048(2.4) | | 5048(2.4) | | 526(2.3) | | 1586(2.0) | |
|  | Missing | | 1728(0.7) | | 2491(0.6) | | 1761(0.8) | | 933(0.6) | | 2729(0.6) | | 1486(0.7) | | 35(0.2) | | 127(0.2) | |
| Usual accommodation | Private (owned or rental) | | 222522(86.3) | | 324440(81.4) | | 202370(87.1) | | 142753(86.4) | | 360438(84.7) | | 184117(85.9) | | 18277(81.0) | | 68097(87.1) | |
|  | Hotel/boarding house/hospital | | 4575(1.8) | | 11066(2.8) | | 3663(1.6) | | 1880(1.1) | | 7676(1.8) | | 4375(2.0) | | 304(1.3) | | 946(1.2) | |
|  | Retirement village | | 24130(9.4) | | 46912(11.8) | | 22261(9.6) | | 18196(11.0) | | 43494(10.2) | | 22086(10.3) | | 2002(8.9) | | 6992(8.9) | |
|  | Residential aged care | | 1510(0.6) | | 4406(1.1) | | 766(0.3) | | 299(0.2) | | 6494(1.5) | | 891(0.4) | | 1435(6.4) | | 1206(1.5) | |
|  | Short term temporary supported | | 3462(1.3) | | 9849(2.5) | | 1490(0.6) | | 1301(0.8) | | 5017(1.2) | | 1523(0.7) | | 496(2.2) | | 844(1.1) | |
|  | Missing | | 1641(0.6) | | 1750(0.4) | | 1716(0.7) | | 724(0.4) | | 2296(0.5) | | 1256(0.6) | | 60(0.3) | | 79(0.1) | |
| Carer availability | Has carer | | 218484(84.7) | | 324513(81.4) | | 198528(85.5) | | 135365(82.0) | | 355361(83.5) | | 184888(86.3) | | 17269(76.5) | | 59951(76.7) | |
|  | Has no carer | | 34037(13.2) | | 59296(14.9) | | 30234(13.0) | | 27982(16.9) | | 57603(13.5) | | 24904(11.6) | | 3687(16.3) | | 16284(20.8) | |
|  | Not applicable | | 3459(1.3) | | 11434(2.9) | | 1802(0.8) | | 830(0.5) | | 9474(2.2) | | 2774(1.3) | | 1576(7.0) | | 1575(2.0) | |
|  | Missing | | 1860(0.7) | | 3180(0.8) | | 1702(0.7) | | 976(0.6) | | 2977(0.7) | | 1682(0.8) | | 42(0.2) | | 354(0.5) | |
| Remoteness | Major City | | 170544(66.1) | | 273572(68.7) | | 153362(66.0) | | 112766(68.3) | | 289084(68.0) | | 137681(64.3) | | 18756(83.1) | | 54687(70.0) | |
|  | Other | | 86108(33.4) | | 122154(30.7) | | 77969(33.6) | | 51925(31.4) | | 134614(31.6) | | 75592(35.3) | | 3643(16.1) | | 22782(29.1) | |
|  | Missing | | 1188(0.5) | | 2697(0.7) | | 935(0.4) | | 462(0.3) | | 1717(0.4) | | 975(0.5) | | 175(0.8) | | 695(0.9) | |
| State | ACT | | 5676(2.2) | | 4178(1.0) | | 5850(2.5) | | 3175(1.9) | | 7389(1.7) | | 2945(1.4) | | 404(1.8) | | 1239(1.6) | |
|  | NSW | | 86813(33.7) | | 137952(34.6) | | 96461(41.5) | | 55148(33.4) | | 143135(33.6) | | 89900(42.0) | | 2434(10.8) | | 20774(26.6) | |
|  | NT | | 995(0.4) | | 683(0.2) | | 535(0.2) | | 939(0.6) | | 1158(0.3) | | 596(0.3) | | 1(<0.01) | | 19(<0.01) | |
|  | QLD | | 46740(18.1) | | 61500(15.4) | | 40609(17.5) | | 29077(17.6) | | 69963(16.4) | | 24626(11.5) | | 884(3.9) | | 14295(18.3) | |
|  | SA | | 21726(8.4) | | 31428(7.9) | | 16756(7.2) | | 11274(6.8) | | 33173(7.8) | | 18341(8.6) | | 1833(8.1) | | 7759(9.9) | |
|  | TAS | | 5807(2.3) | | 13383(3.4) | | 4497(1.9) | | 4533(2.7) | | 13556(3.2) | | 6870(3.2) | | 168(0.7) | | 2162(2.8) | |
|  | VIC | | 61938(24.0) | | 114830(28.8) | | 38317(16.5) | | 40923(24.8) | | 111825(26.3) | | 55335(25.8) | | 11158(49.4) | | 24697(31.6) | |
|  | WA | | 28145(10.9) | | 34469(8.7) | | 29241(12.6) | | 20084(12.2) | | 45216(10.6) | | 15635(7.3) | | 5692(25.2) | | 7219(9.2) | |
| Activity limitations^2^ | Domestic assistance | | 244883(95.0) | | 363330(91.2) | | 224000(96.4) | | 160696(97.3) | | 399530(93.9) | | 203337(94.9) | | 19537(86.5) | | 72230(92.4) | |
|  | Transport | | 238428(92.5) | | 374110(93.9) | | 215159(92.6) | | 151548(91.8) | | 386758(90.9) | | 198386(92.6) | | 21301(94.4) | | 73709(94.3) | |
|  | Meals | | 226334(87.8) | | 351371(88.2) | | 204538(88.1) | | 141627(85.8) | | 362765(85.3) | | 191097(89.2) | | 19003(84.2) | | 68960(88.2) | |
|  | Social and community participation | | 223142(86.5) | | 353934(88.8) | | 200171(86.2) | | 142387(86.2) | | 362605(85.2) | | 185431(86.5) | | 19504(86.4) | | 66066(84.5) | |
|  | Health care tasks | | 211547(82.0) | | 351130(88.1) | | 183104(78.8) | | 124229(75.2) | | 338347(79.5) | | 176672(82.5) | | 20382(90.3) | | 66772(85.4) | |
|  | Home maintenance | | 200776(77.9) | | 287341(72.1) | | 181905(78.3) | | 129175(78.2) | | 323084(75.9) | | 160542(74.9) | | 16193(71.7) | | 57271(73.3) | |
|  | Self-care | | 192313(74.6) | | 331415(83.2) | | 163353(70.3) | | 105187(63.7) | | 296434(69.7) | | 155533(72.6) | | 20714(91.8) | | 70147(89.7) | |
|  | Moving around places | | 168851(65.5) | | 278096(69.8) | | 143756(61.9) | | 90814(55.0) | | 255842(60.1) | | 128786(60.1) | | 17666(78.3) | | 60810(77.8) | |
|  | Movement activities | | 82864(32.1) | | 159169(39.9) | | 55385(23.8) | | 27620(16.7) | | 113895(26.8) | | 53825(25.1) | | 12292(54.5) | | 34123(43.7) | |
|  | Communication | | 55685(21.6) | | 102246(25.7) | | 44861(19.3) | | 26624(16.1) | | 79555(18.7) | | 43621(20.4) | | 4842(21.4) | | 10703(13.7) | |
|  | Other | | 15243(5.9) | | 19854(5.0) | | 15693(6.8) | | 9928(6.0) | | 22953(5.4) | | 14203(6.6) | | 424(1.9) | | 2757(3.5) | |
|  | None | | 521(0.2) | | 774(0.2) | | 365(0.2) | | 163(0.1) | | 936(0.2) | | 365(0.2) | | 75(0.3) | | 286(0.4) | |
| Health conditions | Heart diseases | | 115674(44.9) | | 168319(42.2) | | 99319(42.8) | | 66304(40.1) | | 184190(43.3) | | 87837(41.0) | | 10782(47.8) | | 37139(47.5) | |
|  | Hypertension (high blood pressure) | | 112921(43.8) | | 171356(43.0) | | 104319(44.9) | | 75851(45.9) | | 191644(45.0) | | 92480(43.2) | | 10733(47.5) | | 40623(52.0) | |
|  | Arthritis | | 99066(38.4) | | 140789(35.3) | | 92593(39.9) | | 70351(42.6) | | 169188(39.8) | | 82067(38.3) | | 7071(31.3) | | 28491(36.5) | |
|  | Diseases of the eye | | 65378(25.4) | | 93410(23.4) | | 59639(25.7) | | 43884(26.6) | | 109157(25.7) | | 54355(25.4) | | 4064(18.0) | | 15164(19.4) | |
|  | History of cancer | | 62152(24.1) | | 73723(18.5) | | 46086(19.8) | | 26621(16.1) | | 88412(20.8) | | 37867(17.7) | | 4750(21.0) | | 14083(18.0) | |
|  | Dementia | | 60152(23.3) | | 139273(35.0) | | 58670(25.3) | | 39341(23.8) | | 101725(23.9) | | 66875(31.2) | | 6074(26.9) | | 11023(14.1) | |
|  | Diabetes | | 52403(20.3) | | 73069(18.3) | | 46906(20.2) | | 33113(20.0) | | 85179(20.0) | | 38984(18.2) | | 5149(22.8) | | 17621(22.5) | |
|  | Cerebrovascular diseases | | 49961(19.4) | | 88723(22.3) | | 43943(18.9) | | 29761(18.0) | | 81498(19.2) | | 41333(19.3) | | 5816(25.8) | | 19857(25.4) | |
|  | Chronic lower respiratory diseases | | 46992(18.2) | | 58838(14.8) | | 39364(16.9) | | 26909(16.3) | | 73414(17.3) | | 32018(14.9) | | 3969(17.6) | | 13573(17.4) | |
|  | Osteoporosis | | 44767(17.4) | | 68066(17.1) | | 41353(17.8) | | 31444(19.0) | | 74741(17.6) | | 37845(17.7) | | 3621(16.0) | | 14623(18.7) | |
|  | History of falls | | 43194(16.8) | | 82457(20.7) | | 40720(17.5) | | 26790(16.2) | | 72565(17.1) | | 38516(18.0) | | 5366(23.8) | | 18327(23.4) | |
|  | Deafness/hearing loss | | 42482(16.5) | | 62728(15.7) | | 38388(16.5) | | 26198(15.9) | | 69242(16.3) | | 36240(16.9) | | 2538(11.2) | | 9107(11.7) | |
|  | Depression | | 39441(15.3) | | 62999(15.8) | | 36126(15.6) | | 27807(16.8) | | 64077(15.1) | | 35632(16.6) | | 3493(15.5) | | 11280(14.4) | |
|  | Incontinence | | 38317(14.9) | | 71243(17.9) | | 30089(13) | | 19491(11.8) | | 55590(13.1) | | 29900(14.0) | | 3789(16.8) | | 9845(12.6) | |
|  | Pain | | 35575(13.8) | | 42414(10.6) | | 30305(13.0) | | 22229(13.5) | | 56840(13.4) | | 24900(11.6) | | 2155(9.5) | | 9974(12.8) | |
|  | Kidney & urinary system disorders | | 28123(10.9) | | 37446(9.4) | | 22665(9.8) | | 13586(8.2) | | 42019(9.9) | | 17628(8.2) | | 3025(13.4) | | 9678(12.4) | |
|  | Fracture | | 26601(10.3) | | 48538(12.2) | | 24185(10.4) | | 16941(10.3) | | 45162(10.6) | | 23779(11.1) | | 5139(22.8) | | 25592(32.7) | |
|  | Bedsore | | 14749(5.7) | | 22334(5.6) | | 12312(5.3) | | 7819(4.7) | | 22927(5.4) | | 10864(5.1) | | 1571(7.0) | | 5274(6.7) | |
|  | Malnutrition | | 6075(2.4) | | 9791(2.5) | | 5215(2.2) | | 3463(2.1) | | 9814(2.3) | | 4813(2.2) | | 688(3.0) | | 2220(2.8) | |
|  | Delirium | | 2551(1.0) | | 7561(1.9) | | 1906(0.8) | | 967(0.6) | | 4137(1.0) | | 2283(1.1) | | 893(4.0) | | 2579(3.3) | |
| Assessment Characteristics | |  | |  | |  | |  | |  | |  | |  | |  | |  |
| Year | 2003 | | 2824(1.1) | | 6582(1.7) | | 2442(1.1) | | 1965(1.2) | | 5266(1.2) | | 3290(1.5) | | - | | - | |
|  | 2004 | | 10513(4.1) | | 22595(5.7) | | 10206(4.4) | | 6750(4.1) | | 19109(4.5) | | 11812(5.5) | | - | | - | |
|  | 2005 | | 13368(5.2) | | 28690(7.2) | | 13189(5.7) | | 9477(5.7) | | 24133(5.7) | | 15583(7.3) | | 25(0.1) | | 4(<0.01) | |
|  | 2006 | | 19175(7.4) | | 40343(10.1) | | 17975(7.7) | | 15378(9.3) | | 35113(8.3) | | 21553(10.1) | | 729(3.2) | | 1590(2.0) | |
|  | 2007 | | 22210(8.6) | | 43738(11.0) | | 20597(8.9) | | 18005(10.9) | | 38982(9.2) | | 22870(10.7) | | 2011(8.9) | | 5799(7.4) | |
|  | 2008 | | 27095(10.5) | | 45780(11.5) | | 23952(10.3) | | 18624(11.3) | | 44800(10.5) | | 25325(11.8) | | 2363(10.5) | | 8016(10.3) | |
|  | 2009 | | 30257(11.7) | | 45895(11.5) | | 26449(11.4) | | 19295(11.7) | | 49185(11.6) | | 25757(12.0) | | 2700(12.0) | | 9523(12.2) | |
|  | 2010 | | 28826(11.2) | | 46049(11.6) | | 26315(11.3) | | 19412(11.8) | | 49184(11.6) | | 25248(11.8) | | 3980(17.6) | | 11703(15.0) | |
|  | 2011 | | 35345(13.7) | | 46193(11.6) | | 30916(13.3) | | 22841(13.8) | | 56585(13.3) | | 24733(11.5) | | 4134(18.3) | | 15494(19.8) | |
|  | 2012 | | 42913(16.6) | | 47787(12.0) | | 37580(16.2) | | 22483(13.6) | | 65774(15.5) | | 25028(11.7) | | 4388(19.4) | | 17228(22.0) | |
|  | 2013 | | 25314(9.8) | | 24771(6.2) | | 22645(9.7) | | 10923(6.6) | | 37284(8.8) | | 13049(6.1) | | 2244(9.9) | | 8807(11.3) | |
| Assessors’ professional background ^2^ | Medical | | 121612(47.2) | | 223048(56.0) | | 111180(47.9) | | 66485(40.3) | | 196815(46.3) | | 95639(44.6) | | 19528(86.5) | | 63098(80.7) | |
|  | Nursing | | 198300(76.9) | | 326082(81.8) | | 175305(75.5) | | 121130(73.3) | | 324418(76.3) | | 164924(77.0) | | 20842(92.3) | | 72765(93.1) | |
|  | Health | | 126470(49.0) | | 224788(56.4) | | 108098(46.5) | | 74032(44.8) | | 210512(49.5) | | 99191(46.3) | | 19541(86.6) | | 68673(87.9) | |
|  | Social welfare | | 126234(49.0) | | 221683(55.6) | | 110323(47.5) | | 78206(47.4) | | 211941(49.8) | | 99869(46.6) | | 18269(80.9) | | 53073(67.9) | |
| Service approvals | Home care | | 112685(43.7) | | 309380(77.7) | | 232266(100) | | 165153(100) | | 211601(49.7) | | 119499(55.8) | | 17033(75.5) | | 60632(77.6) | |
|  | Residential care (Permanent) | | 257840(100) | | 398423(100) | | 165903(71.4) | | 89169(54.0) | | 110253(25.9) | | 48179(22.5) | | 5989(26.5) | | 52866(67.6) | |
|  | Respite care | | 228624(88.7) | | 262511(65.9) | | 209114(90.0) | | 135707(82.2) | | 425415(100) | | 214248(100) | | 13523(59.9) | | 56515(72.3) | |
|  | Transition care | | 12421(4.8) | | 23611(5.9) | | 13792(5.9) | | 5617(3.4) | | 20190(4.7) | | 4077(1.9) | | 22574(100) | | 78164(100) | |
|  | Emergency care | | 625(0.2) | | 1458(0.4) | | 595(0.3) | | 215(0.1) | | 436(0.1) | | 1764(0.8) | | 13(0.1) | | 24(<0.01) | |
| Priority^3^ | Between 3 and 14 days | | 125453(48.7) | | 255128(64.0) | | 109206(47.0) | | 72586(44.0) | | 196562(46.2) | | 109020(50.9) | | 19229(85.2) | | 68829(88.1) | |
|  | More than 14 days | | 119412(46.3) | | 110931(27.8) | | 111775(48.1) | | 86361(52.3) | | 207315(48.7) | | 90189(42.1) | | 875(3.9) | | 1526(2.0) | |
|  | Within 48 hours | | 12618(4.9) | | 31529(7.9) | | 10884(4.7) | | 5911(3.6) | | 20763(4.9) | | 14585(6.8) | | 2456(10.9) | | 7776(9.9) | |
|  | Missing | | 357(0.1) | | 835(0.2) | | 401(0.2) | | 295(0.2) | | 775(0.2) | | 454(0.2) | | 14(0.1) | | 33(<0.01) | |

IQR=Interquartile range.

1. Transition care was established 2004-2005, therefore no approvals for 2004 and 2005 are available. Transition care was evaluated within 28 days (and not one year as for other approvals).
2. Missing data: assessors’ professional background (<1.1% for any cell), activity limitation (<0.2% for any cell)
3. Aged Care Assessment Teams respond to referrals for an assessment by allocating a priority category based on the individual’s needs at the time of acceptance for referral.
